# The Canadian HIV and aging cohort study - determinants of increased risk of cardio-vascular diseases in HIV-infected individuals: rationale and study protocol

**DOI:** 10.1186/s12879-017-2692-2

**Published:** 2017-09-11

**Authors:** Madeleine Durand, Carl Chartrand-Lefebvre, Jean-Guy Baril, Sylvie Trottier, Benoit Trottier, Marianne Harris, Sharon Walmsley, Brian Conway, Alexander Wong, Jean-Pierre Routy, Colin Kovacs, Paul A. MacPherson, Kenneth Marc Monteith, Samer Mansour, George Thanassoulis, Michal Abrahamowicz, Zhitong Zhu, Christos Tsoukas, Petronela Ancuta, Nicole Bernard, Cécile L. Tremblay, For the investigators of the Canadian HIV and Aging Cohort Study, Jean-Claude Tardif, Jean-Louis Chiasson, Claude Fortin, Daniel Kaufmann, Gilles Soulez, Guy Cloutier, John Gill, Josée Côté, Julian Falutz, Kenneth Rosenthal, Sophie Bernard, Bastien Lamontagne, Marc Leclerc, Stéphanie Matte, Annie Chamberland, Sylla Mohamed

**Affiliations:** 1 Internal Medicine service, Centre de Recherche du CHUM, Montréal, QC H2J 1T8 Canada; 2 Department of Radiology, Centre hospitalier de l’Université de Montréal, Montréal, Canada; 3 Clinique médicale urbaine du Quartier latin, Montreal, Canada; 4 BC Centre for Excellence in HIV/AIDS, Vancouver, Canada; 5 Division of Infectious Diseases, University Health Network, Toronto, Canada; 6 Infectious Diseases Clinic, Regina Qu’Appelle Health Region, Regina, Canada; 7 Chronic viral infection service and Division of Hematology, McGill University Health Centre, Montreal, Canada; 8 Maple Leaf Medical HIV Research Collaborative Inc., Toronto, Canada; 9 The Ottawa Hospital Research Institute and the University of Ottawa, Ottawa, Canada; 10 Preventive and Genomic Cardiology, McGill University Health Center and Research Institute, Montreal, Canada; 11 Department of Epidemiology and Biostatistics, McGill University, Montreal, Canada; 12 McGill University, Immunology service, Montreal General Hospital, Montreal, Canada; 13 Centre de recherche du CHUM, Montreal, Canada; 14 Research Institute of the McGill University Health Center, Division of Experimental Medicine, McGill University, Division of Clinical Immunology, McGill University health Center (MUHC), Chronic Viral Illness Service, Montreal, Canada

**Keywords:** HIV, Aging, Cardiovascular, Prospective cohort, Study protocol

## Abstract

**Background:**

With potent antiretroviral drugs, HIV infection is becoming a chronic disease. Emergence of comorbidities, particularly cardiovascular disease (CVD) has become a leading concern for patients living with the infection. We hypothesized that the chronic and persistent inflammation and immune activation associated with HIV disease leads to accelerated aging, characterized by CVD. This will translate into higher incidence rates of CVD in HIV infected participants, when compared to HIV negative participants, after adjustment for traditional CVD risk factors. When characterized further using cardiovascular imaging, biomarkers, immunological and genetic profiles, CVD associated with HIV will show different characteristics compared to CVD in HIV-negative individuals.

**Methods/design:**

The Canadian HIV and Aging cohort is a prospective, controlled cohort study funded by the Canadian Institutes of Health Research. It will recruit patients living with HIV who are aged 40 years or older or have lived with HIV for 15 years or more. A control population, frequency matched for age, sex, and smoking status, will be recruited from the general population. Patients will attend study visits at baseline, year 1, 2, 5 and 8. At each study visit, data on complete medical and pharmaceutical history will be captured, along with anthropometric measures, a complete physical examination, routine blood tests and electrocardiogram. Consenting participants will also contribute blood samples to a research biobank. The primary outcome is incidence of a composite of: myocardial infarction, coronary revascularization, stroke, hospitalization for angina or congestive heart failure, revascularization or amputation for peripheral artery disease, or cardiovascular death. Preplanned secondary outcomes are all-cause mortality, incidence of the metabolic syndrome, incidence of type 2 diabetes, incidence of renal failure, incidence of abnormal bone mineral density and body fat distribution. Patients participating to the cohort will be eligible to be enrolled in four pre-planned sub-studies of cardiovascular imaging, glucose metabolism, immunological and genetic risk profile.

**Discussion:**

The Canadian HIV and Aging Cohort will provide insights on pathophysiological pathways leading to premature CVD for patients living with HIV.

**Electronic supplementary material:**

The online version of this article (10.1186/s12879-017-2692-2) contains supplementary material, which is available to authorized users.

## Background

As a result of recent advances in antiretroviral therapy (ART), HIV-infected individuals live longer, with an estimated life expectancy that ranges from 20 to 50 years following infection, and in some cases could reach normal life expectancy [1–3]. Factors that impact on longevity include: the age at infection, the nadir CD4 cell count, the time spent with CD4 counts >500 cells/mm^3^, and other variables [1, 3–8]. In 2015, it was estimated that 50% of HIV-infected persons were >50 years of age, making them susceptible to diseases related to aging [2, 3, 9]. Furthermore, some age-related diseases may be overrepresented in the HIV population, and appear approximately 5 to 10 years earlier than in the general population [10–12]. This has led to the concept of “premature aging” in HIV-infected individuals [13, 14]. This term is controversial, since not all HIV-infected individuals show signs of accelerated aging, and not all components of the aging phenotype have been observed prematurely in the HIV-population. Co-morbid conditions of particular concern include cardiovascular diseases (CVD), the metabolic syndrome (MetS), early onset of osteoporosis, and renal impairment. The pathophysiological processes leading to these phenotypes are complex and not completely understood.

Several hypotheses have been put forward to try to explain premature aging: mitochondrial toxicity, immunodeficiency, antiretroviral toxicity, lifestyle, and a chronic state of immune activation [15–18]. The consequence of chronic immune activation is the development of a senescent immune phenotype, with impaired thymic function, reduced T-cell repertoire and regeneration potential and T cell exhaustion, similarly to what is observed in aging [19, 20].

Large cohort studies have shown an increased risk of CVD in HIV-infected individuals. Compared to age and sex matched controls, we found a hazard ratio for myocardial infarction associated with HIV infection of 2.13 (95% confidence interval [CI] 1.69–2.63) in local data from a Québec administrative database [21]. In the international Data Collection on Adverse Events of anti-HIV Drugs (D:A:D) cohort including 33,347 HIV positive patients, an overall rate of myocardial infarction of 3.48/1000 person-years was reported [22, 23]. A meta-analysis of observational and randomized controlled trials reporting CVD showed that the relative risk of CVD was 1.61 (95% CI 1.43–1.81) among ART-naïve HIV-infected compared with HIV-uninfected people [24, 25].

The overrepresentation of traditional cardiovascular risk factors, particularly smoking, in HIV-infection population can explain part of the excess CVD risk [26]. However, traditional risk factors may not explain the totality of the excess risk. We wish to further characterize the causative pathways and identify predictors of this premature CVD associated with HIV infection. Through better understanding of the particular physiopathology underlying CVD in HIV, our ultimate aim is to prevent negative outcomes and optimize primary care interventions. This study will focus on identifying the type, frequency and determinants of premature CVD in HIV-infected individuals.

### Study hypothesis

The chronic and persistent inflammation associated with HIV disease leads to accelerated aging, characterized by premature CVD, altered metabolism and immune senescence. This will translate into higher incidence rates of CVD in HIV infected participants, when compared to HIV negative participants, after adjustment for traditional CVD risk factors. When characterized further using cardiovascular imaging, biomarkers, immunological and genetic profiles, CVD associated to HIV will show different characteristics compared to CVD in HIV-uninfected individuals.

## Methods/design

### Study design

This is a controlled, prospective cohort study. Pre-planned sub-studies will be nested within the cohort. The exposure of interest for the main cohort and all sub-studies is HIV infection.

### Setting

This is a multicenter study, conducted in 10 Canadian centers.

### Primary outcome

Incidence of CVD, defined as incidence of any of the following conditions: MI, coronary revascularization, stroke, transient ischemic attack, hospitalization for angina or congestive heart failure, revascularization or amputation for peripheral artery disease, or cardiovascular death.

### Secondary outcomes

Secondary outcomes are: i)incidence of all-cause mortality, ii)incidence of major adverse cardiac and cerebrovascular events (MACE - composite outcome composed of cardiovascular death, MI, coronary revascularization or stroke), iii)incidence of individual components of the primary outcome, iv)incidence of the metS, v)incidence of type 2 diabetes, vi)incidence of renal failure, vii)incidence of abnormal bone mineral density and body fat distribution.

Complete definitions of outcomes are available in Additional file 1.

### Study participants

***HIV-infected:*** All individuals living with HIV (as defined by a positive serology for HIV) followed in the HIV clinics of the participating centers, as well as all newly infected or newly diagnosed participants meeting inclusion criteria will be offered to take part into the cohort. ***HIV-uninfected***: HIV-seronegative individuals will be selected from among i)healthy individuals from the general population, reached through publicity campaigns, HIV prevention clinics and participating community members. ii) HIV-uninfected individuals followed at the participating clinics and hospitals. The HIV-negative participants will be frequency matched for age, sex and smoking status, but no individual matching will be done. This will be achieved through periodic comparisons of age and sex distributions of the HIV-positive and HIV-negative participants (every 6 months during the enrollment period) and adjustments in study publicity targeting the HIV-negative group.

### Inclusion criteria

HIV-infected participants must be ≥40 years of age or have HIV infection lasting for at least 15 years (proven by anti-HIV antibody test) at the time of enrollment, be able to provide informed consent and have a life expectancy of at least 1 year. Control group participants must be ≥40 years of age, be able to provide informed consent and have a life expectancy of at least 1 year.

### Interventions and comparisons

This is an observational study, so no study intervention is planned. Participants will receive usual care from their treating physicians throughout the study period.

### Study visits

Patients will be followed yearly for 3 years (baseline, year 1 and year 2), then have additional visits at years 5 and, depending on their recruitment date, at year 8. At each study visit, participants will complete study questionnaires, have a complete medical history and physical examination by a physician, have a panel of blood tests and an electrocardiogram. Twice during the study (year 1 and final year), participants will also undergo a dual energy X-ray absorptiometry to measure bone mineral density. In addition, all participants will be invited to contribute to a research blood bank, to which they will contribute serum and cells. Depending on availability at research sites, participants will also be invited to undergo leukapheresis, to maximize the number of white blood cells available in the biobank. Throughout follow-up, participants’ eligibility to the pre-planned sub-studies will be assessed, and patients will be offered participation in the sub-studies (presented below). Additional files 2 and 3 present tasks performed at each study visit and list of blood tests obtained, respectively.

### Covariates and data collection

At the baseline visit, data will be collected (and entered into the electronical case report form) on socio-demographic factors, all past and current medication, all past and current medical conditions (including high blood pressure, diabetes, dyslipidemia, family history of premature CVD), presence of risk factors for HIV transmission, and lifestyle habits (smoking, alcohol consumption, illicit drug use and level of physical activity). For participants with HIV, date of diagnosis and presumed mode of transmission will also be recorded. A complete physical examination will be performed by the treating physician, and the study nurse will record vital signs, height, weight, and waist circumference.

At the subsequent yearly visits, data on medical history, medication history and lifestyle habits will be updated. Anthropometric measures and physical examination will be repeated. Data on primary and secondary outcomes will be recorded using specific case report forms. Additional file 4 presents the full contents of the case report form that will be filled out at each study visit.

### Statistical considerations

#### Power calculation

Based on data from both our local HIV database and our pilot study [27, 28], we expect the HIV positive population to be 80% male, with a mean age of 50 years at the beginning of the study. Based on data from the Framingham study, expected rates of our primary outcome in HIV-negative participants are 10/1000 person-years for men and 4/1000 person-years for women in the 45–54 age group [29]. The rates more than double for the 55–64 age group [29]. Accordingly, we assume the overall CVD incidence rate in HIV-negative controls will range between 8/1000 PY and 12/1000 PY. Analysis for the primary outcome will be limited to subjects who are free of CVD at baseline. We expect that, at baseline, up 10% of HIV- and 15% of HIV+ subjects will have prevalent CVD [29]. Finally, we assume that an average CVD-free subject will contribute 4 years of follow-up (of the maximum potential follow-up of 5 years). Under these assumptions, the recruitment of 1000 subjects in each group (implying that 900 HIV- and 850 HIV+ subjects will be free of CVD at baseline, contributing 3600 and 3400 PY of follow-up) will yield 80% power with alpha = 0.05 to detect hazard ratios of 1.72 to 1.90 for the primary composite outcomeof CVD. These risk increases are clinically plausible and are actually lower than the more than 2-fold (HR > 2.0) increases in the risk of major CVD events associated with HIV infection [30]. Some studies have reported higher HRs for specific sub-populations or CVD events, e.g. HR 4.0 for risk of stroke among younger subjects [31]. All sample size calculations were done with the PASS software [32].

### Statistical analysis

The analyses will focus on comparing the incidence of CVD in HIV+ subjects and comparing it to the incidence of CVD in HIV- controls.

### Primary outcome

Longitudinal analyses of the CVD incidence/prospective analysis: This analysis will use prospective data and will focus on time until occurrence of the first incident CVD event, as defined by the composite outcome described above. It will be limited to subjects without a history of CVD at cohort entry. Analyses will employ time-to-event statistical methods including Cox proportional hazards (PH) model [33] and its flexible extensions. Time 0 will be defined as the cohort entry and subjects who do not develop CVD during the follow-up will be right-censored at the earliest of times of: loss to follow-up, administrative end of the study or a non-CVD death**.** In all models, the binary indicator of HIV infection will be the main ‘exposure’ and its effect on the risk (hazard) of incident CVD will be adjusted for “standard confounders”: age, sex, ethnicity, education, income, and illicit drug use. However, two different types of models will handle differently the CVD risk factors, to provide a better understanding of the mechanisms through which HIV infection may affect future CVD risks. The first model will adjust the effect of HIV for only the baseline values of CVD risk factors, in addition to “standard confounders”. This will allow us to estimate and test the potential differences in CVD incidence between HIV+ vs. HIV- individuals who had the same initial values of common CVD risk factors, at cohort entry. In contrast, the second model will adjust for the up-dated values of CVD risk factors, modeled by time-varying covariates, representing the most recent value observed until a given date during the follow-up. This model will permit testing if there is a ‘residual’ impact of HIV infection that is *not* mediated through longitudinal changes in common CVD risk factors. The independent association between HIV and CVD incidence will be then estimated through the adjusted HR, with 95% confidence intervals (CI). In both analyses the proportional hazards assumption we will be tested using a flexible extension of the Cox model [34, 35].

### Sensitivity analysis

Retrospective and prospective data analysis: In another model, we will not exclude patients with presence of CVD at baseline. The rationale for this is as follows: if HIV patients experience premature CVD, as we hypothesize, and since HIV patients enter the cohort after varying durations of HIV infection, the risk of CVD associated with HIV might be underestimated if patients with prevalent CVD are excluded from the analysis, i.e.: we would “miss” the earlier events occurring in the HIV infected patients before their entry into the cohort.

In this model, HIV infection will remain the main ‘exposure’, but time 0 will be defined as birth date. HIV infection will be coded in a time-dependent manner: “0” for the time before HIV infection in HIV-infected participants and for HIV-negative controls throughout follow-up, and “1” following first HIV diagnosis in HIV-infected individuals. Incidence of CVD will be modeled using time-to-event statistical methods, as described above, with the first incidence of CVD occurring before cohort entry for patients with prevalent CVD at baseline. Dates of CVD occurrence for patients with prevalent CVD at baseline will be obtained retrospectively (at the baseline visit, through pre-planned data collection for complete medical history). Patients will be right-censored at the earliest of times of: loss to follow-up, administrative end of the study or a non-CVD death**.**

Effect of HIV on the risk (hazard) of incident CVD will be adjusted for “standard confounders”: sex, ethnicity, education level, and income (coded as constant over time), as well as smoking, illicit drug use, sedentary lifestyle, and BMI (coded as time dependent variables). Period of prospective cohort participation (coded in a time-dependent fashion as 0/1) will also be added as a potential confounding variable, as being prospectively followed and assessed for CVD will be likely to increase CVD detection.

### Secondary outcomes

Incidence rates of secondary outcomes will be modeled as described for the primary outcome, through adjusted multivariable survival models.

### Analyses restricted to HIV+ cohort

#### Effect of antiretroviral therapy

To estimate the effect of antiretroviral medication on CVD risk, supplementary analysis will be performed in the cohort of HIV-infected individuals. Incidence rates of the primary outcome will be modeled using survival models similar to those described for the primary outcome, but with drug exposure as the main exposure variable. Drug exposure will be captured as time-dependent variables capturing cumulative exposure, updated for each month of exposure. Models of CVD risk according to exposure to individual drugs will be fitted for abacavir, tenofovir, efavirenz, atazanavir, lopinavir, darunavir, raltegravir, dolutegravir and elvitegravir. These drugs will be analyzed individually due to past signals of cardiovascular toxicity, prevalence of use in our cohort or insufficient data on cardiovascular toxicity available in the literature [21, 36–41]. Exposure to other antiretrovirals will be examined by drug class, as opposed to individually, because we would have insufficient power to detect effect of individual drugs.

### Nadir CD4 and HIV viral load

Lymphocyte CD4 nadir is an important variable in HIV, as it reflects the depth of the immune system suppression by the virus. It can also be used as an indirect proxy for unknown HIV infection date [36]. A survival model will be fitted with nadir CD4 (coded as a continuous variable) as the exposure variable and incidence of CVD as the outcome. Similarly, the cumulative effect of unsuppressed viral replication will be analysed, as it has been shown to be correlated with mortality in HIV infection [42]. Unsuppressed viral load leads to immune activation and inflammation, which, according to our research hypothesis, lead to CVD. In a survival model, the time-dependent, cumulative area under the curve of viral load measurements will be used as the exposure variable, with incidence of CVD as the outcome.

### Pre-planned sub-studies

#### Sub study 1 - characterization of atherosclerotic plaque

Lo reported increased prevalence of subclinical coronary atherosclerotic disease in HIV [43]. It is yet unclear if the composition of the atherosclerotic plaque in HIV is similar to that seen in the general population [36, 43–45]. More data are needed to characterize the atherosclerotic process, with possible implications for screening and treatment. In this sub-sub-study, 5 cardiac imaging modalities will be applied to characterize the atherosclerosis in HIV positive patients and compare it to that in HIV-negative controls: 1) positron emitting tomography (PET) scan of the ascending aorta and carotid arteries 2)carotid arteries ultrasound with measurement of intima-media thickness 3) cardiac computed tomography scan without injection of contrast media, 4) cardiac computed tomography scan with injection of contrast media, and 5) intravascular coronary ultrasound (in patients for whom coronary angiogram is clinically indicated). Most imaging modalities will be cross-sectional, but cardiac computed tomography scan and carotid arteries ultrasound will be repeated to assess progression of coronary artery disease.

### Hypothesis

Atherosclerotic plaque has a different composition in HIV infected individuals, which can be described by imaging modalities.

### Outcomes

For each of the imaging modalities, the primary outcome is difference in atherosclerotic plaque presence and composition between HIV+ and HIV-. For repeated measures (cardiac scan with contrast media), the outcomes include rate of progression of total plaque volume.

### Population and setting

Participants recruited in the Montreal study sites will be eligible to this sub-study. Participants with a moderate Framingham risk score (ranging from 5 to 20%) will be asked to take part in this sub-study. Computed tomography scans, carotid ultrasound and vascular PET scans will be done at the Centre Hospitalier de l’Université de Montréal radiology core lab. Coronary angiograms and intravascular ultrasound will be done at the CHUM coronary catheter laboratory. Projected sample sizes have been calculated individually for each imaging modalities to ensure sufficient power to detect clinically meaningful differences. We expect to perform 30 cardiac PET scans, 200 intima-media measurements, 200 non-contrast media cardiac scans, 190 contrast-media cardiac scans, and 100 intravascular ultrasounds.

#### Sub-study 2-characterization of dysglycemia

There are conflicting results in the literature regarding relative prevalence and determinants of dysglycemia (defined as impaired fasting glucose, impaired glucose tolerance or diabetes) in HIV-infected patients compared to the HIV-negative population [46–49]. Antiretrovirals, lipodystrophy, and altered metabolism due to long-lasting inflammation could contribute to development of dysglycemia in this population [49, 50]. In this sub-study, oral glucose tolerance tests will be used to assess patients at risk of or with known dysglycemia in a prospective fashion, with levels of biomarkers to further characterize the nature of HIV-associated dysglycemia.

### Hypothesis

Dysglycemia in HIV infection is associated with a different metabolic profile. Precisely, we hypothesize there will be more endothelial dysfunction (as measured by increased ICAM1, VCAM1, and E-selectin), more coagulation activation (as measured by increased PAI-1 and fibrinogen), more oxidative stress (as measured by oxidized-LDL), and more inflammation (as measured by IL-6, TNF-α, CRP) in HIV+ than in HIV- patients with dysglycemia.

### Outcomes

The primary outcome is difference in area under the curve for the biomarkers throughout the oral glucose tolerance test between HIV+ and HIV- patients.

### Population and settings

All patients from the main cohort recruited in the Montréal sites with fasting plasma glucose > = to 5.6 mmol/l or HbA1c > = 5.6% will be asked to undergo an oral glucose tolerance test to screen for dysglycemia. Serum will be banked during the oral glucose tolerance tests, and patients with abnormalities in glucose metabolism will have their sample analyzed for the biomarkers of interest (see hypothesis). Measurements will be done in the metabolism core lab of the CHUM. According to the incidence of dysglycemia, we aim to perform about 150 oral glucose tolerance tests, and to fully analyse serum for biomarkers in 1/3 (50). This will grant 90% power at alpha 0.05 to detect a 10% difference in oxidized LDL between the HIV + and HIV- patients, with similar power for the other biomarkers.

#### Sub-study 3-characterization of immune profile in CVD

Globally impaired immunity secondary to HIV infection might contribute to the risk of CVD in HIV infected individuals via several pathways. Of particular interest is the depletion of Th17 lymphocytes, leading to microbial translocation from the gut, sustained immune activation, and eventually immune senescence [51–55]. In this sub-study, we will perform cross sectional immunological studies to better characterize the contribution of various immune profiles on development of CVD, and how those differ between HIV+ and HIV- individuals.

### Hypothesis

CVD associated with HIV is immunologically distinct from CVD in HIV uninfected patients: impaired immunity induced by HIV infection leads to an immune risk profile that contributes to development of premature CVD.

### Outcomes

Immunological analyses will focus on Th17 paucity, monocyte activation, Cytomegalovirus infection, and presence of an immune risk phenotype characterised by T-cell senescence and immune activation. The primary outcomes are specific to each of those three aspects. They involve measures of correlations between immune profiles and total coronary plaque volume, in participants with and without HIV.

### Population and setting

For this sub study, the presence of CVD will be measured as total coronary plaque volume, which is measured on injected cardiac Computed tomography (CT) scan. Banked samples from all patients who undergo injected cardiac CT scan will be analyzed. The planned sample size is 190 patients in total.

#### Sub-study 4 - characterization of genetic profile in CVD

Several studies have reported that a genetic risk score is associated with incident and prevalent CVD [56–58]. It remains unknown whether a genetic predisposition for atherosclerosis could also potentiate HIV-specific mechanisms for CVD. We propose to evaluate whether a genetic risk score comprised of 30 single nucleotide polymorphisms (already validated in genome-wide association studies) can predict the presence of CVD, and whether the risk score is more strongly associated with CVD in HIV+ as compared to HIV- individuals.

### Hypothesis

A genetic risk score will be more strongly associated with subclinical CVD in HIV+ than in HIV- participants.

### Outcomes

The primary outcome is the difference in risk scores between HIV+ and HIV- patients with CVD.

### Population and settings

The genetic risk score will be measured in all patients from the prospective cohort.

For sub-studies 1–4, details of the planned statistical analysis are presented in Additional file 5 ***.***

## Discussion

The Canadian HIV and Aging Cohort Study is a large, controlled, prospective cohort. It aims to recruit 1000 participants living with HIV, and 1000 participants unexposed to HIV. While its primary endpoints are significant clinical events for patients living with HIV, the pre-planned sub-studies will increase our basic understanding of the complex interplay between the immune alterations caused by chronic HIV infection and CVD. The cohort will provide a strong base for future studies of comorbidities associated with aging with HIV.

Several methodological aspects of this cohort warrant further discussion. First, the age at enrolment of the participants living with HIV was carefully considered. As our main focus was cardiovascular health, recruiting participants who were too young would have led to low event rates and low power. Yet recruiting patients with HIV infection that is long-lasting might result in missing the early alterations of the cardiovascular system that are driven by the chronic state of inflammation found in those patients. We settled for an enrolment age of 40 years or older, but planned that patients with very long-lasting HIV infection (15 years or more) could be recruited even if younger than 40.

Selection of the HIV uninfected patients was another key aspect of our design. One strategy would have been to simply recruit patients from the general population through publicity campaigns, with a frequency matching for age and sex only. However, very large differences in traditional cardiovascular risk factors, particularly smoking, may have obscured the risk due to HIV, so we decided to frequency match the selection on smoking status as well. Also, as HIV infection is associated with certain lifestyles, which in turn could impact on the outcomes of our study, we elected to recruit our HIV uninfected cohort as well from clinics that treated our patients living with HIV. Those choices imply that our results will not be generalizable to the general population, but we felt that they suited our general aim to isolate the effect of HIV infection as much as possible.

Power calculations were based on event rates from the Framingham cohort [29]. These rates may not apply to the Canadian population, and may be lower now that aggressive treatment of cardiovascular risk factors is widespread. As such, we may lack power to detect differences in the occurrence of the primary endpoint. However, our power calculations were conservative, aiming to be able to detect HRs for our composite endpoint of 1.72 to 1.90, which is lower than the 2.13 95%CI [1.69–2.63] we observed in our own population [59].

Our study encompasses the entire research spectrum, from collection and analysis of epidemiological data on lifestyle, quality of life and medical co-morbidities to advanced fundamental imaging and immunological research. We have access to detailed epidemiological data, and a biobank with stored cells and plasma. Our team of experts also spans the whole research continuum from clinical to basic science, including community members, health practitioners, specialists in cardiology, immunology, virology, genetics, radiology, and internal medicine, with a balance between clinical and basic scientists. This study structure will allow rapid and in-depth investigations into novel immuno-metabolic pathways that lead to CVD in the setting of HIV infection.

Another important advantage of our cohort setting is the universal health care system that is available to all participants of our study. This will greatly contribute to alleviate discrepancies in healthcare that would be due to socioeconomic status. Central data collection and management is another strength. Overall, flexibility of the study structure, with possible addition of further sub-studies to the main cohort, is a major capacity-building aspect of this cohort.

We believe the Canadian HIV and Aging Cohort Study will be a powerful research tool to offer rapid responses to emerging clinical questions for people living with HIV and their healthcare providers.

## Additional files

Additional File 1: Study Outcomes definitions. (DOCX 34 kb)

Additional File 2: Flow chart of tasks performed at each study visits. (DOCX 23 kb)

Additional File 3: Blood tests to be drawn at each study visit. (DOCX 26 kb)

Additional file 4: Detailed data collection form for the Canadian HIV and Aging Study. (PDF 830 kb)

Additional File 5: Supplementary Statistical Analysis for sub studies. (DOCX 24 kb)

## Abbreviations

AIDS: Acquired Immunodeficiency Syndrome
CVD: Cardiovascular Disease
HIV: Human Immunodeficiency Virus
MetS: Metabolic Syndrome
MI: Myocardial Infarction
