# Supplementary material for: The Canadian HIV and aging cohort study - determinants of increased risk of cardio-vascular diseases in HIV-infected individuals: rationale and study protocol
Source: BMC Infect Dis. 2017 Sep 11;17:611. doi: 10.1186/s12879-017-2692-2 (PMC5594495; doi:10.1186/s12879-017-2692-2)
Supplement: Supplementary file 1 — Study Outcomes definitions. (DOCX 34 kb) [file 12879_2017_2692_MOESM1_ESM.docx]

**Additional File 1 – Details of outcomes definitions**

| Event | Definition |
| --- | --- |
| Components of the primary outcome | |
| Myocardial infarction | Defined according to the American Heart Association as a rise and fall of cardiac biomarker values (preferably cardiac troponin) with at least one value above the 99th percentile upper reference limit and with at least one of the following: 1)symptoms of ischemia 2)new or presumed new significant ST-segment-T wave changes or new left bundle-branch block 3)development of pathological Q waves on ECG 4)Imaging evidence of new loss of viable myocardium or new regional wall motion abnormality 5)Identification of an intracoronary thrombus by angiography or autopsy. All five sub-types of myocardial infarction are included as outcomes. [60] |
| Coronary revascularization | Coronary artery bypass graft surgery or percutaneous coronary revascularization. |
| Stroke | Sudden onset of focal neurological deficit in a location consistent with the territory of a major cerebral artery, categorized as ischaemic, hemorrhagic or unspecified |
| Transient ischemic attack | Defined according to the American Heart Association/American Stroke association as a transient episode of neurological dysfunction caused by focal brain, spinal cord, or retinal ischemia, without acute infarction[61]. To be considered as an outcome in our study, the TIA must be considered as a TIA by a trained neurologist. |
| Hospitalization for cardio-vascular disease | Defined as an emergency care visit or hospitalization for symptoms attributable to angina by a trained cardiologist, with or without changes on electrocardiography, with or without elevation of cardiac biomarkers OR emergency care visit or hospitalization for congestive heart failure as evaluated by a trained cardiologist. |
| Amputation or revascularization for peripheral artery disease | Surgical bypass or endarterectomy (including aortic aneurysm repair), endovascular dilatation or stenting or amputation due to peripheral artery disease. |
| Cardiovascular death | Sudden cardiac death (a sudden and unexpected pulseless condition attributable to cessation of cardiac mechanical activity [29]), fatal myocardial infarction or death from heart failure. |
| Secondary outcomes | |
| Metabolic Syndrome | The metabolic syndrome is defined as per the International Diabetes Federation by the presence of central obesity (waist circumference >= 94 cm for Caucasian men and >= 80 cm for Caucasian women, or ethnicity specific values for other groups) AND any 4 of the following 4 diagnostic measures: Elevated triglycerides (>1.7 mmol/L or drug treatment for elevated triglycerides), reduced high-density lipoprotein (HDL) cholesterol (< 0.9 mmol/L in men, <1.1 mmol/L in women, or drug treatment for reduced HDL cholesterol), elevated blood pressure (>130 mm Hg systolic blood pressure, >85 mm Hg diastolic blood pressure, or drug treatment for or previously diagnosed hypertension), and elevated fasting glucose (>5.6 mmol/l or drug treatment for elevated glucose) or previously diagnosed type 2 diabetes. [62] |
| Type 2 diabetes | Type 2 diabetes will be defined according to the Canadian Diabetes Association as two of the following abnormalities measured on different days: 1)Fasting (no caloric intake for at least 8 hours) plasma glucose >= 7.0 mmol/L, 2)casual plasma glucose >= 11.1 mmol/l with symptoms of diabetes (Casual = any time of the day, without regard to the interval since the last meal, classic symptoms of diabetes = polyuria, polydipsia and unexplained weight loss), 3)2h plasma glucose in a 75-g oral glucose tolerance test >=11.1 mmol/L or 4) HBA1c values >=6.5%.[63] |
| Renal Failure | Renal failure is defined according to creatinine clearance (in ml/min/1,73m^2^) calculated by the CKD-EPI formula, as defined by the Kidney Disease Outcomes Quality Initiative (KDOQI), as stage 1; CrCl> 90 n, stage 2; CrCl 60-89, stage 3; 30-59, stage 4; 15-29 and stage 5; <15 or renal replacement therapy.  Renal failure stages will be entered according to the creatinine measured at the study visit, using the CKD-EPI formula (For women with creatinine <= 62umol/L : GFR = 144 x (Scr/62)^-0,329^ x (0.993)^Age^ , for women with creatinine > 62umol/L : GFR = 144 x (Scr/62)^-1,209^ x (0.993)^Age^ For men with creatinine <= 80umol/L : DFG = 141 x (Scr/80)^-0,411^ x (0.993)^Age^ , for men with creatinine > 80umol/L : DFG = 141 x (Scr/80)^-1,209^ x (0.993)^Age^ , for participant of black race, multiply result by 1,159.[64]) |
| Osteoporosis and osteopenia | Osteoporosis is defined as T score less than 2.5 on bone osteodensitometry, and osteopenia as T-score less than 1.5 on bone osteodensitometry. |
