# Supplementary material for: The Canadian HIV and aging cohort study - determinants of increased risk of cardio-vascular diseases in HIV-infected individuals: rationale and study protocol
Source: BMC Infect Dis. 2017 Sep 11;17:611. doi: 10.1186/s12879-017-2692-2 (PMC5594495; doi:10.1186/s12879-017-2692-2)
Supplement: Supplementary file 2 — Flow chart of tasks performed at each study visits. (DOCX 23 kb) [file 12879_2017_2692_MOESM2_ESM.docx]

**Additional File 2 – Flow chart of tasks performed at each study visits**

| **Flow chart of tasks performed at each study visits** | | | | | | |
| --- | --- | --- | --- | --- | --- | --- |
| **Study Procedures** | **Screening and enrolment visits** | **Year 1**^a^ | **Year 2**  **(between Y2 & Y3)** ^a^ | **Year 5**^a^ | **Year 8** |  |
| Get Informed consent^b^ | X |  |  |  |  |  |
| Baseline data collection  Demographics  Complete medical history  Family medical history  Cardiovascular risk factors  Complete medication history | X |  |  |  |  |  |
| Take vital signs and measurements:  Blood pressure  Heart rate  Respiratory rate  Temperature  Height  Weight  Waist circumference | X | X | X | X | X |  |
| Current medications (list all medication taken) | X | X | X | X | X |  |
| Fill Psychosocial Questionnaire | X | X | X | X | X |  |
| Collect data on primary and secondary outcomes |  | X | X | X | X |  |
| Blood work ^c^ | X | X | X | X | X |  |
| Assess eligibility to take part to sub-studies | X | X | X |  |  |  |
| Get Informed consents for eligible sub-studies | X | X | X |  |  |  |
| Schedule ECG (within 90 days) | X | X | X | X | X |  |
| Schedule DEXA scan (within 365 days) | X |  |  | X |  |  |
| Physician visit, including complete history and physical examination, within +/- 90 days. | X | X | X | X | X |  |
| ^a^ Visits must take place within 90 days of yearly visit planned date.  ^b^ See appendix 2 for list of ICFs to be filled at each visit  ^c^ See appendix 3 for list of blood work to be drawn at each visit | | | | | |  |
