# Supplementary material for: The Canadian HIV and aging cohort study - determinants of increased risk of cardio-vascular diseases in HIV-infected individuals: rationale and study protocol
Source: BMC Infect Dis. 2017 Sep 11;17:611. doi: 10.1186/s12879-017-2692-2 (PMC5594495; doi:10.1186/s12879-017-2692-2)
Supplement: Supplementary file 3 — Blood tests to be drawn at each study visit. (DOCX 26 kb) [file 12879_2017_2692_MOESM3_ESM.docx]

**Additional File 3 – Details of blood tests to be drawn at each study visit**

| **Blood test (blood volume)** | **Screening** | **Y1** | **Y2 (between Y2 and 3)** | **Y5** | **Y8** |
| --- | --- | --- | --- | --- | --- |
| **Complete blood count (4 ml)** | x | x | x | x | x |
| **Sedimentation rate (4ml)** | x | x | x | x | x |
| **Biochemistry (including fasting blood glucose, urea, creatinine, complete electrolytes, LFTs, TG, LDL, HDL, albumin, (4 ml)** | x | x | x | x | x |
| **Immunology (CD4, CD8) (4 ml)** | x | x* | x* | x* | x* |
| **Viral Load (8 ml)** | x | x* | x* | x* | x* |
| **Syphilis (EIA-RPR) (5 ml)** | x | x | x | x | x |
| **Toxoplasmosis serology, IgG IgM (5 ml)** | x |  |  |  |  |
| **CMV serology IgG IgM (5 ml)** | x | x | x | x | x |
| **HSV serology HSV-1 HSV-2 IgG and IgM (5 ml)** | x |  |  |  |  |
| **HAV serology (5ml)** | x |  |  |  |  |
| **HBsAg, Anti-HBs, Anti-HBc (5 ml)** | x |  |  |  |  |
| **Anti-HCV (15ml)** | X |  |  |  |  |
| **TSH (5ml)** | x | x | x | x | x |
| **HbA1c (5ml)** | x | X | X | X | X |
| **hs CRP (5ml)^a^** | x | x | x | x | x |
| **Free testosterone, SHBG, total testosterone (5 ml)** | x |  |  |  |  |
| **Insulin (5 ml)** | x | x | x | x | x |
| **25(OH) vitamin D (5ml)** | x |  |  |  |  |
| **Folic acid (5 ml)** | x |  |  |  |  |
| **Apo lipoprotein A (5 ml) ^a^** | x | x | x | x | x |
| **Apo lipoprotein B (5 ml) ^a^** | x | x | x | x | x |
| **troponin I (5 ml)** | x | x | x | x | x |
| **pro-BNP (5 ml) ^a^** | x | x | x | x | x |
| **Fibrinogen, D-Dimers, INR, PTT (4 ml) ^a^** | x |  |  |  |  |
| **Factor VII (4 ml) ^a^** | x |  |  |  |  |
| **Von Villebrand factor (12 ml)^a^** | x |  |  |  |  |
| **Anti-thrombin III (12 ml)^a^** | x |  |  |  |  |
| **urinaryn albumin/creatinine ratio** | x | x | x | x | x |
| **urinary protein/creatinine ratio** | x | x | x | x | x |
| **Urine Analysis** | x | x | x | x | x |
| **Total blood volume (ml/table spoons)** | **161/11** | **66/4** | **66/4** | **66/4** | **66/4** |
|  | **Baseline** | **Y1 lab** | **Y2 Lab** | **Y5 Lab** | **Y8 Lab** |
| **Research Blood bank (110 ml)** | x | *** | *** | X | x |
| **Total blood volume (ml/tablespoons)** | **110 / 7** | **110 /7** | **110 /7** | **110 /7** | **110 / 7** |
| * Only in HIV-positive **Only in HIV-negative at risk for HIV infection, to be done by treating physician **^a^** Optional, only if available at study site labs do not need to be repeated if performed within 3 months of baseline visit  *** For Montreal Sites Only: Research banking will be performed each year only for subjects participating in the cardiovascular imaging sub-studies and for subjects who developed the primary outcome.(Myocardial infarction, coronary revascularization, stroke, hospitalization for cardiovascular cause, amputation or revascularization for peripheral arterial disease). | | | | | |
