# Supplementary material for: The Canadian HIV and aging cohort study - determinants of increased risk of cardio-vascular diseases in HIV-infected individuals: rationale and study protocol
Source: BMC Infect Dis. 2017 Sep 11;17:611. doi: 10.1186/s12879-017-2692-2 (PMC5594495; doi:10.1186/s12879-017-2692-2)
Supplement: Supplementary file 4 — Detailed data collection form for the Canadian HIV and Aging Study. (PDF 830 kb) [file 12879_2017_2692_MOESM4_ESM.pdf]

Annotated Study Book for Study Design: The Canadian HIV and Aging Cohort - Determinanats of increased risk of cardio-vascular diseases in HIV-infected individuals

Study Design Version: 0.0

Protocol: CTN272

CTN272 Study Design

Generated by Central Designer ™

November 27, 2014 4:33PM

The Canadian HIV and Aging Cohort - Determinanats of increased risk of cardio-vascular diseases in HIV-infected individuals: System Screening (Screening) [frm_SysScr]		
**Initials [sct_Initials]**		
1.	Initials<br>[Initials]	**[itm_Initials]**
2.	Screening Date<br>[Screening Date]	**[itm_ScreeningDate]**<br>/ /

The Canadian HIV and Aging Cohort - Determinanats of increased risk of cardio-vascular diseases in HIV-infected individuals: System Enrollment (Enrollment) [frm_SysEnr]		
**Subject Number [sct_SubjectNum]**		
1.	Subject ID<br>[Subject ID]	**[itm_SubjectID]**

**The Canadian HIV and Aging Cohort - Determinanats of increased risk of cardio-vascular diseases in HIV-infected individuals: Date of Visit (DOV) [frm_DOV]**		
**Date of Visit [sct_DateofVisit]**		
1.	Date of Report<br>[Date of Report]	**[itm_DateofReport]**<br>/ /

**The Canadian HIV and Aging Cohort - Determinanats of increased risk of cardio-vascular diseases in HIV-infected individuals: Participant Identification (Subject Identification) [frm_SubjectID]**		
**Participant Identification [frm_SubjectID]**		
1.	Subject ID<br>[Subject ID]	**[itm_SubjectID]**
2.	Initials<br>[Initials]	**[itm_Initials]**

The Canadian HIV and Aging Cohort - Determinanats of increased risk of cardio-vascular diseases in HIV-infected individuals: Inclusion Exclusion Fulfillment (IncExc Fullfillment) [frm_IncExc_Fulfill]		
**Inclusion Exclusion Fulfillment [frm_IncExc_Fulfill]**		
1.	Consent Date<br>[Consent Date]	**[itm_ConsentDate]**<br>/ /
2.	Does the subject fulfill all the Inclusion and Exclusion Criteria as specified in the protocol?<br>[All Criteria met?]	**[itm_Fulfill]**<br>Yes No
3.	Is the subject enrolled based on the decision made by a physician?<br>[Decision by the Physician?]	**[itm_PISigned]**<br>Yes No

The Canadian HIV and Aging Cohort - Determinanats of increased risk of cardio-vascular diseases in HIV-infected individuals: Inclusion & Exclusion (IncExc Deviation) [frm_IncExc]

	Criterion	Ctireria Number
1. ✓		

Criteria Entry [sct_Criteria]

1.1	Criterion<br>[Criterion]	**[itm_Criterion]**<br>○ Inclusion ○ Exclusion
1.2	Criteria Number<br>[Ctireria Number]	**[itm_CriteriaNum]**<br>**[cod_CriteriaNum]**

The Canadian HIV and Aging Cohort - Determinanats of increased risk of cardio-vascular diseases in HIV-infected individuals: Demographics (Demographics) [frm_Dem]		
**Demographics [frm_Dem]**		
1.	Date of Birth<br>[Date of Birth]	**[itm_DOB]**<br>/ /
2.	Sex<br>[Sex]	**[itm_Sex]**<br>Male Female Irretrievable
3.	Place of Birth<br>[Place of Birth]	**[itm_PlaceofBirth]**<br>Canada<br>**[itm_CountryofOrigin]**<br>Other<br>Country of Origin<br>**[itm_YearResidentofCanada]**<br>What year did subject became resident of Canada? (4-digit integer)<br>Irretrievable
4.	Race<br>[Race]	**[itm_Race]**<br>**[cod_Race]**
5.	Source of income<br>[Source of income]	**[itm_SourceIncome]**<br>Remunerated Employment Unemployment Insurance Welfare Other Irretrievable
6.	Annual Income<br>[Annual Income]	**[itm_AnnualIncome]**<br>**[cod_AnnualIncome]**
7.	Highest level of education completed<br>[Highest level of education completed]	**[itm_Education]**<br>**[cod_Education]**
8.	Marital Housing Status<br>[Marital Housing Status]	**[itm_MaritalHousing]**<br>**[cod_MaritalHousing]**

The Canadian HIV and Aging Cohort - Determinanats of increased risk of cardio-vascular diseases in HIV-infected individuals: Sub-Study Enrollment (Sub-Study Enrollment) [frm_Sub_Enroll]		
**Sub-Study Enrollment [frm_Sub_Enroll]**		
1.	Whether the subject is eligible to enrolled in any sub-studies?<br>[Eligible in any sub-studies?]	**[itm_SubStudy_Enroll]**<br>◯Yes ◯No
2.	Which sub-study is the subject enrolled?<br>[Which sub-study is the subject enrolled?]	**[itm_SubStudy_Choice]**<br>☐Cadiovascular Imaging sub-study ☐Metabolic syndrome sub-study

The Canadian HIV and Aging Cohort - Determinanats of increased risk of cardio-vascular diseases in HIV-infected individuals: Risk -- HIV Risk Factor (Risk -- HIV Risk Factor) [frm_Risk_HIVRiskFactor]		
**Risk -- HIV Risk Factor [frm_Risk_HIVRiskFactor]**		
1.	HIV Risk Factor<br>[HIV Risk Factor]	**[itm_HIVRiskFactor]**<br>☐ Homosexual (MSM)<br>☐ Heterosexual<br>☐ Bisexual<br>☐ Hemophilia<br>☐ Originating from Endemic Region<br>☐ Bisexual Transmission<br>☐ Homosexual Transmission<br>☐ Heterosexual Transmission<br>☐ Mother to Infant Transmission<br>☐ Intravenous Drug User (IDU)<br>☐ Injection (piercing, tattooing)<br>☐ Transfusion<br>☐ Prostitution<br>☐ Other or Unknown<br>☐ Irretrievable

The Canadian HIV and Aging Cohort - Determinanats of increased risk of cardio-vascular diseases in HIV-infected individuals: Risk -- CVD Risk Smoking Status (Risk -- CVD Risk Smoking Status) [frm_Risk_CVD_SStatus]		
**Risk -- CVD Risk Smoking Status [frm_Risk_CVD_SStatus]**		
1.	Have You Ever Smoked?<br>[Ever Smoked?]	**[itm_SmokingStatus]**<br>◯ Never Smoked ◯ Current or Ex Smoker ◯ Irretrievable

The Canadian HIV and Aging Cohort - Determinanats of increased risk of cardio-vascular diseases in HIV-infected individuals: Risk -- CVD Risk Smoking (Risk -- CVD Risk Smoking) [frm_Risk_CVD_S]

Risk -- CVD Risk Smoking [frm_Risk_CVD_S]

1.	Current or Ex Smoker<br>At what age did you start smoking? (2-digit integer)<br>[At what age did you start smoking?]	**[itm_SmokingStartAge]**
2.	Number of Cigarettes per Day (1 pack=25) (2-digit integer)<br>[Number of Cigarettes per Day (1 pack=25)]	**[itm_NumberCigarettes]**
3.	Are you current or ex smoker?<br>[Current or Ex Smoker?]	**[itm_SmokingClassification]**<br>○ Current Smoker<br>○ **[itm_SmokingStopAge]**<br>Ex Smoker<br>At what age did you stop smoking? (2-digit integer)

The Canadian HIV and Aging Cohort - Determinanats of increased risk of cardio-vascular diseases in HIV-infected individuals: Risk -- CVD Risk Drinking Status (Risk -- CVD Risk Drinking Status) [frm_Risk_CVD_AStatus]		
**Risk -- CVD Risk Drinking Status [frm_Risk_CVD_AStatus]**		
1.	Have You Ever Drank?<br>[Ever Drank?]	**[itm_AlcoholStatus]**<br>○ Does not Drink ○ Current or Ex Drinker ○ Irretrievable

**The Canadian HIV and Aging Cohort - Determinanats of increased risk of cardio-vascular diseases in HIV-infected individuals: Risk -- CVD Risk Drinking (Risk -- CVD Risk Drinking) [frm_Risk_CVD_A]**		
**Details [sct_Alcohol]**		
1.	Current or Ex Drinker<br>Types of drinker<br>(Social Drinker: drinks alcohol without excessive consumption, psycho social or health problem related to alcohol)<br>(Excessive drinker: social or medical problem related to alcohol consumption, alcoholism, or excessive alcohol consumption)<br>*(Excessive alcohol consumption: Man of more than 14 consumption per week and Woman of more than 9 consumption per week)*<br>*[Types of drinker]*	**[itm_AlcoholClassification]**<br>○ Ex Drinker ○ Social Drinker ○ Excessive Drinker
2.	Number of Alcohol Units per Week (3-digit integer)<br>(Beer 1 glass (750ml)=1 unit, Wine 1 glass=1 unit, Liquor 1 shot or measure (about 1.5 oz)=1 unit)<br>[Number of Alcohol Units per Week]	**[itm_NumberofAlcohol]**

**The Canadian HIV and Aging Cohort - Determinanats of increased risk of cardio-vascular diseases in HIV-infected individuals: Risk -- CVD Risk Drug Use Status (Risk -- CVD Risk Drug Use Status) [frm_Risk_CVD_DStatus]**		
**Risk -- CVD Risk Drug Use Status [frm_Risk_CVD_DStatus]**		
1.	Does the subject use drug (in the past or present)?<br>[Does the subject use drug (in the past or present)?]	**[itm_UseDrug]**<br>◯Yes ◯No ◯Irretrievable

The Canadian HIV and Aging Cohort - Determinanats of increased risk of cardio-vascular diseases in HIV-infected individuals: Risk -- CVD Risk Drug (Risk -- CVD Risk Drug) [frm_Risk_CVD_D]

	Drug	Mode	Age Started	Ongoing
1. ✓				

Details Entry [sct_DrugDetails]

If yes, please fill out the details here.

1.1	Drug<br>[Drug]	**[itm_DrugTypes]**<br>◯ Cocaine or Crack<br>◯ Heroine or Opiates<br>◯ Stimulants<br>◯ **[itm_Other_Spec]**<br>Other<br>Specify
1.2	Mode<br>[Mode]	**[itm_DrugMode]**<br>◯ Ingestion ◯ Inhalation ◯ Injection ◯ Unknown
1.3	Age Started (2-digit integer)<br>[Age Started]	**[itm_DrugStart]**
1.4	Ongoing<br>[Ongoing]	**[itm_DrugOngoing]**<br>◯ Yes<br>◯ **[itm_DrugStopped]**<br>No<br>Age Stopped (2-digit integer)

The Canadian HIV and Aging Cohort - Determinanats of increased risk of cardio-vascular diseases in HIV-infected individuals: Risk -- CVD Waist Circumference Fram Risk Score (Risk -- CVD Waist Circumference Fram Risk Score) [frm_Risk_CVDWaistFram]		
**Risk -- CVD Waist Circumference Fram Risk Score [frm_Risk_CVDWaistFram]**		
1.	Waist Circumference (3-digit integer)<br>[Waist Circumference]	**[itm_WaistCir]**<br>cm[b]
2.	Fram Risk Score	**[itm_FramRiskScore]**

The Canadian HIV and Aging Cohort - Determinanats of increased risk of cardio-vascular diseases in HIV-infected individuals: Medical History -- HIV (MH -- HIV) [frm_MH_HIV]		
**Medical History -- HIV [frm_MH_HIV]**		
1.	Know date of diagnosis?<br>[Know date of diagnosis?]	**[itm_Know_DOD]**<br>**[itm_DateofDiagnosis]**<br>Yes<br>Date of Diagnosis<br>/ /<br>**[itm_YearHIV]**<br>No<br>Estimated year of HIV infection (4-digit integer)
2.	Date CD4 Nadir<br>[Date CD4 Nadir]	**[itm_DateCD4Nadir]**<br>/ /
3.	CD4 % (2-digit integer)<br>[CD4 %]	**[itm_CD4Percent]**
4.	Abs. (cells/ mm3) (4-digit integer)<br>[Abs. (cells/ mm3)]	**[itm_CD4Abs]**

The Canadian HIV and Aging Cohort - Determinanats of increased risk of cardio-vascular diseases in HIV-infected individuals: Medical History -- HIV Positive (MH -- HIV Positive) [frm_MH_HIV_Positive]		
**Medical History -- HIV Positive [frm_MH_HIV_Positive]**		
1.	HIV +<br>[HIV +]	**[itm_HIVPositive]**<br>◯Yes ◯No

The Canadian HIV and Aging Cohort - Determinanats of increased risk of cardio-vascular diseases in HIV-infected individuals: Medical Histry -- CVD Risk Factors (MH -- CVD Risk Factors) [frm_MH_CVDRisk]

Medical Histry -- CVD Risk Factors [frm_MH_CVDRisk]

1.	Does the subject have the following?<br>[Does the subject have the following?]	**[itm_DiseaseRisk]**<br>☐ **[itm_YearDiabetes]**<br>Diabetes<br>Since when (year)? (4-digit integer)<br>☐ **[itm_YearHBP]**<br>Hight Blood Pressure<br>Since when (year)? (4-digit integer)<br>☐ Family history of premature CVD<br>(in 1st degree relative under 50 in man or under 60 in woman) *Fill the section below*<br>☐ Family hisotry of CVD<br>(in 1st degree relative at any age)<br>☐ **[itm_RegularPAFreq]**<br>Practice regular physical activity<br>Frequency<br>○ At least 30-m every day<br>○ At least 30-m 3 times a week<br>○ At least 30-m 1 time a week<br>○ Irretrievable<br>☐ **[itm_RecallWeight]**<br>Recall his/her weight at age 20 or current weight if under 20<br>Weight (3-digit integer)<br>kg[b]<br>☐ Irretrievable

	Which Relative?	What Event?	At What Age?
2. ✓			

Family Premature CVD Entry [sct_FamilyPrematureCVD]

2.1	Which Relative?<br>[Which Relative?]	**[itm_Relative]**<br>**[cod_Relative]**
2.2	What Event?<br>[What Event?]	**[itm_Event]**
2.3	At What Age? (2-digit integer)<br>[At What Age?]	**[itm_Age]**

The Canadian HIV and Aging Cohort - Determinanats of increased risk of cardio-vascular diseases in HIV-infected individuals: Vtal Signs (Vital Signs) [frm_VitalSign]		
**Vtal Signs [frm_VitalSign]**		
1.	Vital Signs Items<br>[Vital Signs Items]	**[itm_VSItems]**<br>☐ **[itm_Weight]**<br>Weight (1 decimal place) ____ kg[b]<br>☐ **[itm_Height]**<br>Height (1 decimal place) ____ cm[b]<br>☐ **[itm_Temperature]**<br>Temperature (1 decimal place) ____ C[b]<br>☐ **[itm_Pulse]**<br>Pulse (3-digit integer) ____ bpm[b]<br>☐ **[itm_BP]**<br>Enter blood pressure (sitting): **[SystolicVariable]** **[DiastolicVariable]**<br>____ / ____ mmHg

The Canadian HIV and Aging Cohort - Determinanats of increased risk of cardio-vascular diseases in HIV-infected individuals: Physical Exam (Physical Exam) [frm_PE]		
**Physical Exam [frm_PE]**		
1.	Was Physical Examination Normal?<br>[Was Physical Examination Normal?]	**[itm_NoYes_PE]**<br>Yes No Not Done
2.	Is there any change from baseline?<br>(If change from screening, document it on AE page.)<br>[Change from baseline?]	**[itm_Change_PE]**<br>Yes No Not Applicable

The Canadian HIV and Aging Cohort - Determinanats of increased risk of cardio-vascular diseases in HIV-infected individuals: Physical Abnormalities (Physical Abnormalities) - Repeating Form [frm_PE_Abnormalities]

#	Record No	System	PE Abnormalities
1			

Physical Abnormalities [frm_PE_Abnormalities]		
1.	Record No.<br>[Record No]	**[itm_RowPE]**
2.	Sytem<br>[System]	**[itm_Cat_PE]**<br>◯ General appearance<br>◯ ENT<br>◯ Eyes<br>◯ Head and neck<br>◯ Respiratory<br>◯ Cardiovascular<br>◯ Abdomen<br>◯ Genitourinary<br>◯ Anorectal<br>◯ Extremities<br>◯ **[itm_OtherSpec_PE]** Other Specify:<br>◯ Musculoskeletal<br>◯ Lymph nodes<br>◯ Neurological<br>◯ Psychiatric<br>◯ Endocrine<br>◯ Hematopoietic<br>◯ Other
3.	Physical Abnormalities<br>[PE Abnormalities]	**[itm_PEAbnorm]**

The Canadian HIV and Aging Cohort - Determinanats of increased risk of cardio-vascular diseases in HIV-infected individuals: Lab Hematology (Hematology) [frm_LabHemat]

Lab Hematology [frm_LabHemat]

1.	Sample Collection Date<br>[Sample Collection Date]	**[itm_LabDate]**<br>/ /

Lab Hematology [sct_LabHemat]

# ✔	Lab Category	Item Number	Tests	Result	Units
2.a	Hematology	1	Hemoglobin		G/L
2.b	Hematology	2	Hematocrit		N/A
2.c	Hematology	3	WBC		xE9/L
2.d	Hematology	4	RBC		xE12/L
2.e	Hematology	5	MCV		f/L
2.f	Hematology	6	Platelets		xE9/L
2.g	Hematology	7.1	Neutrophils		xE9/L
2.h	Hematology	7.2	Neutrophils		%
2.i	Hematology	8.1	Lymphoctyes		xE9/L
2.j	Hematology	8.2	Lymphoctyes		%
2.k	Hematology	9.1	Monocytes		xE9/L
2.l	Hematology	9.2	Monocytes		%
2.m	Hematology	10.1	Eosinophils		xE9/L
2.n	Hematology	10.2	Eosinophils		%
2.o	Hematology	11.1	Basophils		xE9/L
2.p	Hematology	11.2	Basophils		%
2.q	Hematology	12	ESR		mm/h
2.r	Hematology	13	TPO (anti-thyroidiens)		UI/mL
2.s	Hematology	14	TG (anti-thyroglobuline)		N/A
2.t	Hematology	15	Fibrinogen		G/L
2.u	Hematology	16	INR		Ratio
2.v	Hematology	17	D_Dimere		ug/L
2.w	Hematology	18	Von Willebrand Factor		U/mL
2.x	Hematology	19	Antithrombin III		U/mL
2.y	Hematology	20	Factor VII		U/mL

Lab Hematology Entry [sct_LabHemat]

2.1	Lab Category<br>[Lab Category]	**[itm_LabCat]**<br>**[cod_LabCat]**
2.2	Item Number<br>[Item Number]	**[itm_Item_Number]**<br>**[cod_Row]**
2.3	Lab Test Names<br>[Tests]	**[itm_LabTest_Hema]**<br>**[cod_LabTest_Hema]**
2.4	Result<br>[Result]	**[itm_LabResult]**<br>**[itm_LabResultTxt]** Sign *(Fill in -, +, <, >, etc. Leave blank if not applicable.)*<br>**[itm_LabResultNum]** Value *(Fill in a numeric value. Leave blank if not applicable.)*
2.5	Units<br>[Units]	**[itm_LabUnit]**<br>**[cod_Units]**

The Canadian HIV and Aging Cohort - Determinanats of increased risk of cardio-vascular diseases in HIV-infected individuals: Lab Chemistry (Chemistry) [frm_LabChem]

Lab Chemistry [frm_LabChem]

1.	Sample Collection Date<br>[Sample Collection Date]	**[itm_LabDate]**<br>/ /
2.	Was the subject fasting?<br>[Was the subject fasting?]	**[itm_LabFasting]**<br>Yes No Not Applicable

Lab Chemistry [sct_LabChemistry]

#	Lab Category	Item Number	Tests	Result	Units
3.a	Chemistry	1	Glucose		mmol/L
3.b	Chemistry	2	Creatinine		umol/L
3.c	Chemistry	3	Creatinine Clearnace		mL/Min (ML/M)
3.d	Chemistry	4	Sodium		mmol/L
3.e	Chemistry	5	Potassium		mmol/L
3.f	Chemistry	6	Chloride (Chlorure)		mmol/L
3.g	Chemistry	7	Magnesium		mmol/L
3.h	Chemistry	8	Phosphorus		mmol/L
3.i	Chemistry	9	Calcium		mmol/L
3.j	Chemistry	10	Albumin		G/L
3.k	Chemistry	11	Total Bilirubin		umol/L
3.l	Chemistry	12	Total CO2		mmol/L
3.m	Chemistry	13	AST		U/L
3.n	Chemistry	14	ALT		U/L
3.o	Chemistry	15	GGT		U/L
3.p	Chemistry	16	Alkaline Phosphatase		U/L
3.q	Chemistry	17	LDH		U/L
3.r	Chemistry	18	CK		U/L
3.s	Chemistry	19	Amylase		U/L
3.t	Chemistry	20	Lipase		U/L
3.u	Chemistry	21	Triglyceride		mmol/L
3.v	Chemistry	22	Total Cholesterol		mmol/L
3.w	Chemistry	23	HDL - Cholesterol		mmol/L
3.x	Chemistry	24	LDL- Cholesterol		mmol/L
3.y	Chemistry	25	Ratio Cholesterol/HDL		Ratio
3.z	Chemistry	26	Apolipoprotein A1		G/L
3.aa	Chemistry	27	Apolipoprotein B		G/L
3.ab	Chemistry	28	Insulin (Insuline)		pmol/L
3.ac	Chemistry	29	TSH		mUI/L
3.ad	Chemistry	30	25(OH) Vit D		nmol/L
3.ae	Chemistry	31	Total Testosterone		nmol/L
3.af	Chemistry	32	Free Testosterone		pmol/L
3.ag	Chemistry	33	hs- CRP		mg/L
3.ah	Chemistry	34	Folic Acid		nmol/L
3.ai	Chemistry	35	Troponin T (HS)		ng/L
3.aj	Chemistry	36	Triglyceride		%

Lab Chemistry Entry [sct_LabChemistry]

3.1	Lab Category<br>[Lab Category]	**[itm_LabCat]**<br>**[cod_LabCat]**
3.2	Item Number<br>[Item Number]	**[itm_Item_Number]**<br>**[cod_Row]**
3.3	Lab Test Names<br>[Tests]	**[itm_LabTest_Chem]**<br>**[cod_LabTest_Chem]**
3.4	Result<br>[Result]	**[itm_LabResult]**<br>**[itm_LabResultTxt]**<br>Sign *(Fill in -, +, <, >, etc. Leave blank if not applicable.)*<br>**[itm_LabResultNum]**<br>Value *(Fill in a numeric value. Leave blank if not applicable.)*
3.5	Units<br>[Units]	**[itm_LabUnit]**<br>**[cod_Units]**

The Canadian HIV and Aging Cohort - Determinanats of increased risk of cardio-vascular diseases in HIV-infected individuals: Lab Urinalysis (Urinalysis) [frm_LabUrine]

Lab Urinalysis [frm_LabUrine]

1.	Sample Collection Date<br>[Sample Collection Date]	**[itm_LabDate]**<br>/ /

Lab Urinalysis [sct_LabUrin_Urin]

# ✔	Lab Category	Item Number	Test Names	Result	Units
2.a	Urine	1	Specific Gravity		N/A
2.b	Urine	2	Glucose		N/A
2.c	Urine	3	pH		N/A
2.d	Urine	4	Hemoglobin/Blood		N/A
2.e	Urine	5	Ketones		N/A
2.f	Urine	6	Protein		N/A
2.g	Urine	7	Leukocyte		N/A

Lab Urinalysis Entry [sct_LabUrin_Urin]

2.1	Lab Category<br>[Lab Category]	**[itm_LabCat]**<br>**[cod_LabCat]**
2.2	Item Number<br>[Item Number]	**[itm_Item_Number]**<br>**[cod_Row]**
2.3	Lab Test Names<br>[Test Names]	**[itm_LabTest_Urin]**<br>**[cod_LabTest_Uri]**
2.4	Result<br>[Result]	**[itm_LabResult]**<br>**[itm_LabResultTxt]**<br>Sign *(Fill in -, +, <, >, etc. Leave blank if not applicable.)*<br>**[itm_LabResultNum]**<br>Value *(Fill in a numeric value. Leave blank if not applicable.)*
2.5	Units<br>[Units]	**[itm_LabUnit]**<br>**[cod_Units]**

Lab Urine Urinary Albumin/Creatinin Ratio [sct_LabUrin_Ratio]

# ✔	Lab Category	Item Number	Test Names	Result	Units
3.a	Urine	1	Albumin/ creatinine ratio		mg/mL
3.b	Urine	2	Prot U\ creatinine		mg/mL

Lab Urine Urinary Albumin/Creatinin Ratio Entry [sct_LabUrin_Ratio]

3.1	Lab Category<br>[Lab Category]	**[itm_LabCat]**<br>**[cod_LabCat]**
3.2	Item Number<br>[Item Number]	**[itm_Item_Number]**<br>**[cod_Row]**
3.3	Lab Test Names<br>[Test Names]	**[itm_LabTest_Urin]**<br>**[cod_LabTest_Uri]**
3.4	Result<br>[Result]	**[itm_LabResult]**<br>**[itm_LabResultTxt]**<br>Sign *(Fill in -, +, <, >, etc. Leave blank if not applicable.)*<br>**[itm_LabResultNum]**<br>Value *(Fill in a numeric value. Leave blank if not applicable.)*
3.5	Units<br>[Units]	**[itm_LabUnit]**<br>**[cod_Units]**

The Canadian HIV and Aging Cohort - Determinanats of increased risk of cardio-vascular diseases in HIV-infected individuals: Lab Urine Microscopic Exam (Urine Microscopic Exam) [frm_LabUrine_Micro]

Lab Urine Microscopic Exam [frm_LabUrine_Micro]

1.	Sample Collection Date<br>[Sample Collection Date]	**[itm_LabDate]**<br>/ /

Lab Microscopic Exam [sct_LabUrin_Micro]

# ✔	Lab Category	Item Number	Test Names	Result	Units
2.a	Urine	9	WBC		N/A
2.b	Urine	10	RBC		N/A
2.c	Urine	11	EPITH		N/A
2.d	Urine	12	CASTS (GRAN)		N/A
2.e	Urine	13	CASTS (HYALINE)		N/A
2.f	Urine	14	BACTERIA		N/A
2.g	Urine	15	CRYSTALS		N/A
2.h	Urine	16	Other		N/A

Lab Microscopic Exam Entry [sct_LabUrin_Micro]

2.1	Lab Category<br>[Lab Category]	**[itm_LabCat]**<br>**[cod_LabCat]**
2.2	Item Number<br>[Item Number]	**[itm_Item_Number]**<br>**[cod_Row]**
2.3	Lab Test Names<br>[Test Names]	**[itm_LabTest_Urin]**<br>**[cod_LabTest_Uri]**
2.4	Result<br>[Result]	**[itm_LabResult]**<br>**[itm_LabResultTxt]**<br>Sign *(Fill in -, +, <, >, etc. Leave blank if not applicable.)*<br>**[itm_LabResultNum]**<br>Value *(Fill in a numeric value. Leave blank if not applicable.)*
2.5	Units<br>[Units]	**[itm_LabUnit]**<br>**[cod_Units]**

	Other, specify
3. ✔	

Lab Urin Microscopic Exam Other Findings Entry [sct_LabUrin_Micro_Other]

3.1	Other<br>Specify<br>[Other, specify]	**[itm_Other_Spec]**

The Canadian HIV and Aging Cohort - Determinanats of increased risk of cardio-vascular diseases in HIV-infected individuals: Lab Immunology (Immunology) [frm_LabImmu]		
**Lab Immunology [frm_LabImmu]**		
1.	Sample Collection Date<br>[Sample Collection Date]	**[itm_LabDate]**<br>/ /
2.	WBC (x E9/L)<br>[WBC (x E9/L)]	**[itm_LabWBC]**
3.	Lymphocytes<br>[Lymphocytes]	**[itm_LabLymph]**
4.	Were subsets measured?<br>[Were subsets measured?]	**[itm_SubMeasured]**<br>Yes No

The Canadian HIV and Aging Cohort - Determinanats of increased risk of cardio-vascular diseases in HIV-infected individuals: Lab Immunology Subsets (Immunology Subsets) [frm_LabImmu_Sub]

Lab Immunology Subsets [sct_LabImmu_Sub]

# ✔	Lab Category	Item Number	Test	Result	Units
1.a	Immunology	1	CD3+		%
1.b	Immunology	1	CD3+		xE9/L
1.c	Immunology	2	CD4+		%
1.d	Immunology	2	CD4+		xE9/L
1.e	Immunology	3	CD8+		%
1.f	Immunology	3	CD8+		xE9/L
1.g	Immunology	4	CD8 + 38		%
1.h	Immunology	4	CD8 + 38		xE9/L
1.i	Immunology	5	CD19		%
1.j	Immunology	5	CD19		xE9/L
1.k	Immunology	6	CD16 + 56		%
1.l	Immunology	6	CD16 + 56		xE9/L
1.m	Immunology	7	CD8 + 38 Fluor.Rel.		%
1.n	Immunology	7	CD8 + 38 Fluor.Rel.		xE9/L
1.o	Immunology	8	Ratio CD4 / CD8		%
1.p	Immunology	8	Ratio CD4 / CD8		xE9/L

Lab Immunology Subsets Entry [sct_LabImmu_Sub]

1.1	Lab Category<br>[Lab Category]	**[itm_LabCat]**<br>**[cod_LabCat]**
1.2	Item Number<br>[Item Number]	**[itm_Item_Number]**<br>**[cod_Row]**
1.3	Test Category<br>[Test]	**[itm_LabTest_Immu]**<br>**[cod_LabTest_Immu]**
1.4	Result<br>[Result]	**[itm_LabResult]**<br>**[itm_LabResultTxt]**<br>Sign *(Fill in -, +, <, >, etc. Leave blank if not applicable.)*<br>**[itm_LabResultNum]**<br>Value *(Fill in a numeric value. Leave blank if not applicable.)*
1.5	Units<br>[Units]	**[itm_LabUnit]**<br>**[cod_Units]**

The Canadian HIV and Aging Cohort - Determinanats of increased risk of cardio-vascular diseases in HIV-infected individuals: Lab Virology (Virology) [frm_LabViro]

Lab Virology [frm_LabViro]

1.	Sample Collection Date<br>[Sample Collection Date]	**[itm_LabDate]**<br>/ /
2.	HIV-RNA Viral Load<br>[HIV-RNA Viral Load]	**[itm_HIVRNAViralLoad]**<br>< Lower limit of quantification recorded below Value recorded below > Upper limit of quantification recorded below
3.	Type of assay<br>[Type of assay]	**[itm_TypeAssay]**<br>**[cod_TypeAssay]**

Lab Virology [sct_LabViro]

# ✔	Lab Category	Item Number	Test Category	Result
4.a	Virology	1	Copies per mL	
4.b	Virology	2	HIV-RNA (Log) value	

Lab Virology Entry [sct_LabViro]

4.1	Lab Category<br>[Lab Category]	**[itm_LabCat]**<br>**[cod_LabCat]**
4.2	Item Number<br>[Item Number]	**[itm_Item_Number]**<br>**[cod_Row]**
4.3	Test Category<br>[Test Category]	**[itm_LabTest_Viro]**<br>Copies per mL HIV-RNA (Log) value
4.4	Result<br>[Result]	**[itm_LabResult]**<br>**[itm_LabResultTxt]**<br>Sign *(Fill in -, +, <, >, etc. Leave blank if not applicable.)*<br>**[itm_LabResultNum]**<br>Value *(Fill in a numeric value. Leave blank if not applicable.)*

The Canadian HIV and Aging Cohort - Determinanats of increased risk of cardio-vascular diseases in HIV-infected individuals: HLAB* 5701 (HLAB* 5701) [frm_LabHLAB5701]

HLAB* 5701 [frm_LabHLAB5701]

1.	Sample Collection Date<br>[Sample Collection Date]	**[itm_LabDate]**<br>/ /

HLAB* 5701 [sct_HLAB5701]

# ✔	Lab Category	Item Number	Tests	Result
2.a	HLAB 5701	1	HLAB* 5701	

HLAB* 5701 Entry [sct_HLAB5701]

2.1	Lab Category<br>[Lab Category]	**[itm_LabCat]**<br>**[cod_LabCat]**
2.2	Item Number<br>[Item Number]	**[itm_Item_Number]**<br>**[cod_Row]**
2.3	Lab Test Names<br>[Tests]	**[itm_LabTest_HLAB5701]**<br>○ HLAB* 5701
2.4	Result<br>[Result]	**[itm_LabResult]**<br>**[itm_LabResultTxt]**<br>Sign *(Fill in -, +, <, >, etc. Leave blank if not applicable.)*<br>**[itm_LabResultNum]**<br>Value *(Fill in a numeric value. Leave blank if not applicable.)*

The Canadian HIV and Aging Cohort - Determinanats of increased risk of cardio-vascular diseases in HIV-infected individuals: Lab Co-Infections (Co-infections) [frm_LabCoInfections]

Lab Co-Infections [frm_LabCoInfections]

1.	Sample Collection Date<br>[Sample Collection Date]	**[itm_LabDate]**<br>/ /

Lab Immunology /Virology [sct_LabCoI]

Please fill in + as Positive, - as Negative in the field of "Sign" and leave the "Value" field blank.

# ✔	Lab Category	Item Number	Test	Test Details	Result
2.a	Co-Infections	1.1	Hepatitis A	Total anti-HAV VHA	
2.b	Co-Infections	1.2	Hepatitis A	Anti HAV (VHA) IgM	
2.c	Co-Infections	2.1	Hepatitis B	HBsAg	
2.d	Co-Infections	2.2	Hepatitis B	Anti HBc	
2.e	Co-Infections	2.3	Hepatitis B	Anti HBs	
2.f	Co-Infections	2.4	Hepatitis B	HBV-DNA (Quantitatif)	
2.g	Co-Infections	3.1	Hepatitis C	Anti-HCV	
2.h	Co-Infections	3.2	Hepatitis C	RNA- HCV Qualitative	
2.i	Co-Infections	3.3	Hepatitis C	RNA-HCV Quantitative	
2.j	Co-Infections	3.4	Hepatitis C	HCV (Genotype)	
2.k	Co-Infections	4.1	Cytomegalovirus (CMV)	IgM	
2.l	Co-Infections	4.2	Cytomegalovirus (CMV)	IgG	
2.m	Co-Infections	5.1	Toxoplasmosis	IgM	
2.n	Co-Infections	5.2	Toxoplasmosis	IgG	
2.o	Co-Infections	6.1	Syphilis	EIA (screening)	
2.p	Co-Infections	6.2	Syphilis	RPR (follow-up)	
2.q	Co-Infections	7	Gonorrhea	Not Applicable	
2.r	Co-Infections	8	Condyloma (human papillomavirus)	Not Applicable	
2.s	Co-Infections	9	Chlamydia	Not Applicable	
2.t	Co-Infections	10	Anti-HSV 1 (oral herpes type 1)	Not Applicable	
2.u	Co-Infections	11	Anti-HSV II (oral herpes type 2)	Not Applicable	
2.v	Co-Infections	12	Candida	Not Applicable	

Lab Immunology /Virology Entry [sct_LabCoI]

Please fill in + as Positive, - as Negative in the field of "Sign" and leave the "Value" field blank.

2.1	Lab Category<br>[Lab Category]	**[itm_LabCat]**<br>**[cod_LabCat]**
2.2	Item Number<br>[Item Number]	**[itm_Item_Number]**<br>**[cod_Row]**
2.3	Test Category<br>[Test]	**[itm_LabTest_CoI]**<br>**[cod_LabTest_CoI]**
2.4	Test Details<br>[Test Details]	**[itm_LabTest_CoI_Details]**<br>**[cod_LabResult_CoIDetails]**
2.5	Result<br>[Result]	**[itm_LabResult]**<br>**[itm_LabResultTxt]**<br>Sign *(Fill in -, +, <, >, etc. Leave blank if not applicable.)*<br>**[itm_LabResultNum]**<br>Value *(Fill in a numeric value. Leave blank if not applicable.)*

The Canadian HIV and Aging Cohort - Determinanats of increased risk of cardio-vascular diseases in HIV-infected individuals: Lab Test Sub-Study (Sub-Study Test) [frm_Lab_SubStudy]		
**Lab Test Sub-Study [frm_Lab_SubStudy]**		
1.	What sub-study test has been done?<br>[What sub-study test has been done?]	**[itm_LabTestSubStudy]**<br>☐ Cardiac TEP scan<br>☐ Carotid ultrasound<br>☐ Non-Contrast-Cardiac CT<br>☐ IVUS<br>☐ Oral Glucose Tolerance Test<br>☐ Mixed Meal<br>☐ Leukapheresis<br>☐ Genetics

The Canadian HIV and Aging Cohort - Determinanats of increased risk of cardio-vascular diseases in HIV-infected individuals: Lab EKG (EKG) [frm_LabEKG]		
**Lab EKG [frm_LabEKG]**		
1.	Sample Collection Date<br>[Sample Collection Date]	**[itm_LabDate]**<br>/ /
2.	EKG Result<br>[DexaScan Result Other]	**[itm_LabResult_EKG]**

The Canadian HIV and Aging Cohort - Determinanats of increased risk of cardio-vascular diseases in HIV-infected individuals: Lab Dexa Scan (Dexa Scan) [frm_LabDexaScan]

Lab Dexa Scan [frm_LabDexaScan]

1.	Sample Collection Date<br>[Sample Collection Date]	**[itm_LabDate]**<br>/ /
2.	DexaScan Result *(please record other findings here)*<br>*[DexaScan Result Other]*	**[itm_LabResult_DexaScan]**

T-scores [sct_LabTscores]

# ✔	Lab Category	Item Number	Tests	Result
3.a	Bone Mineral Density T-Scores	1	L1-L4	
3.b	Bone Mineral Density T-Scores	2	Femoral Neck	
3.c	Bone Mineral Density T-Scores	3	HipTotal	

T-scores Entry [sct_LabTscores]

3.1	Lab Category<br>[Lab Category]	**[itm_LabCat]**<br>**[cod_LabCat]**
3.2	Item Number<br>[Item Number]	**[itm_Item_Number]**<br>**[cod_Row]**
3.3	Lab Test Names<br>[Tests]	**[itm_LabTest_Tscores]**<br>◯ L1-L4 ◯ Femoral Neck ◯ HipTotal
3.4	Result<br>[Result]	**[itm_LabResult]**<br>**[itm_LabResultTxt]**<br>Sign *(Fill in -, +, <, >, etc. Leave blank if not applicable.)*<br>**[itm_LabResultNum]**<br>Value *(Fill in a numeric value. Leave blank if not applicable.)*

Body Fat Distribution [sct_BodyFatDistri]

# ✔	Lab Category	Item Number	Tests	Result	Units
4.a	Body Fat Distribution	1	Legs		%
4.b	Body Fat Distribution	2	Trunk		%
4.c	Body Fat Distribution	3	Total		%

Body Fat Distribution Entry [sct_BodyFatDistri]

4.1	Lab Category<br>[Lab Category]	**[itm_LabCat]**<br>**[cod_LabCat]**
4.2	Item Number<br>[Item Number]	**[itm_Item_Number]**<br>**[cod_Row]**
4.3	Lab Test Names<br>[Tests]	**[itm_LabTest_BodyFatDistri]**<br>◯ Legs ◯ Trunk ◯ Total
4.4	Result<br>[Result]	**[itm_LabResult]**<br>**[itm_LabResultTxt]**<br>Sign *(Fill in -, +, <, >, etc. Leave blank if not applicable.)*<br>**[itm_LabResultNum]**<br>Value *(Fill in a numeric value. Leave blank if not applicable.)*
4.5	Units<br>[Units]	**[itm_LabUnit]**<br>**[cod_Units]**

The Canadian HIV and Aging Cohort - Determinanats of increased risk of cardio-vascular diseases in HIV-infected individuals: IVUS (Sub-study -- IVUS) [frm_Sub_IVUS]

IVUS [frm_Sub_IVUS]

1.	Date patient had exam<br>[Date patient had exam]	**[itm_ExamDate]**<br>/ /

	Plaque Number	IVUS Anatomical Site	Plaque Volume	Result (integer)	Result (integer)	Result (integer)	Result (integer)
2. ✓							

IVUS Result Entry [sct_IVUS_Result]

2.1	Plaque Number<br>[Plaque Number]	**[itm_Plaque_Number]**<br>**[cod_Row]**
2.2	IVUS Anatomical Site<br>[IVUS Anatomical Site]	**[itm_IVUS_Anatomical]**<br>○ Left main ○ IVA ○ Circumflex ○ Right
2.3	Plaque Volume<br>[Plaque Volume]	**[itm_PlaqueVol]**<br>**[itm_Sub_Result_PlaqueVol]** Plaque Volume Result (integer) **[itm_LabUnit_mm3]** ○ mm3
2.4	Percent Fibrous Result (integer)<br>[Result (integer)]	**[itm_Sub_Result_Fibrous]**
2.5	Percent Fibro-fatty Result (integer)<br>[Result (integer)]	**[itm_Sub_Result_Fibrofatty]**
2.6	Percent Necrotic Core Result (integer)<br>[Result (integer)]	**[itm_Sub_Result_NecroticCore]**
2.7	Percent Dense-calcium Result (integer)<br>[Result (integer)]	**[itm_Sub_Result_DenseCa]**

The Canadian HIV and Aging Cohort - Determinanats of increased risk of cardio-vascular diseases in HIV-infected individuals: Sub-study -- Carotid Ultrasound (Sub-study -- Carotid Ultrasound) [frm_Sub_CarotidUltra]

Sub-study -- Carotid Ultrasound [frm_Sub_CarotidUltra]

1.	Date patient had exam<br>[Date patient had exam]	**[itm_ExamDate]**<br>/ /
2.	Able to perform exam (technically impossible)<br>[Able to perform exam (technically impossible)]	**[itm_ExamAble]**<br>Yes No

Carotid Ultrasound Result [sct_CUltrasound_Result]

# ✔	Item Number	Carotid Ultrasound Categories	Result (2 decimal places)	Units
3.a		Carotid intima-media thickness		mm
3.b		Carotid intima thickness		mm
3.c		Carotid media thickness		mm

Carotid Ultrasound Result Entry [sct_CUltrasound_Result]

3.1	Item Number<br>[Item Number]	**[itm_Item_Number]**<br>**[cod_Row]**
3.2	Carotid Ultrasound Categories<br>[Carotid Ultrasound Categories]	**[itm_CUltrasound_Cat]**<br>Carotid intima-media thickness Carotid intima thickness Carotid media thickness
3.3	Result (2 decimal places)<br>[Result (2 decimal places)]	**[itm_Sub_Result_Float]**
3.4	Units<br>[Units]	**[itm_LabUnit]**<br>**[cod_Units]**

The Canadian HIV and Aging Cohort - Determinanats of increased risk of cardio-vascular diseases in HIV-infected individuals: Sub-study -- Contrast Cardiac CT (Sub-study -- Contrast Cardiac CT) [frm_Sub_ContrastCT]

Sub-study -- Contrast Cardiac CT [frm_Sub_ContrastCT]

1.	Date patient had exam<br>[Date patient had exam]	**[itm_ExamDate]**<br>/ /
2.	Presence of significant stenosis<br>[Presence of significant stenosis]	**[itm_Stenosis]**<br>Yes No
3.	Was another radiological or diagnostic test ordered following this test (according to the results or as per recommendation of the radiologist)?<br>[Was another radiological or diagnostic test ordered?]	**[itm_2ndTestOrdered]**<br>**[itm_2ndTest]** Yes<br>Additional test was related to heart condition? Heart Condition Non-heart Condition<br>No

Contrast Cardiac CT Result [sct_ContrastCT_Result]

# ✔	Item Number	Contrast Cardiac CT Categories	Result (integer)	Units
4.a	1	Percent stenosis Left Main		N/A
4.b	2	Percent stenosis interventricular artery		N/A
4.c	3	Percent stenosis circumflex artery		N/A
4.d	4	Percent stenosis right coronary artery		N/A
4.e	5	Total low-attenuation plaque volume		mm3

Contrast Cardiac CT Result Entry [sct_ContrastCT_Result]

4.1	Item Number<br>[Item Number]	**[itm_Item_Number]**<br>**[cod_Row]**
4.2	Contrast Cardiac CT Categories<br>[Contrast Cardiac CT Categories]	**[itm_ContrastCT_Cat]**<br>Percent stenosis Left Main / Percent stenosis interventricular artery / Percent stenosis circumflex artery / Percent stenosis right coronary artery / Total low-attenuation plaque volume
4.3	Result (integer)<br>[Result (integer)]	**[itm_Sub_Result_Integer]**
4.4	Units<br>[Units]	**[itm_LabUnit]**<br>**[cod_Units]**

The Canadian HIV and Aging Cohort - Determinanats of increased risk of cardio-vascular diseases in HIV-infected individuals: Sub-study -- Non-contrast cardiac CT (Sub-study -- Non-contrast cardiac CT) [frm_Sub_NonContrastCT]

Sub-study -- Non-contrast cardiac CT [frm_Sub_NonContrastCT]

1.	Date patient had exam<br>[Date patient had exam]	**[itm_ExamDate]**<br>/ /
2.	Was another radiological or diagnostic test ordered following this test (according to the results or as per recommendation of the radiologist)?<br>[Was another radiological or diagnostic test ordered?]	**[itm_2ndTestOrdered]**<br>○ **[itm_2ndTest]** Yes<br>Additional test was related to heart condition?<br>○ Heart Condition<br>○ Non-heart Condition<br>○ No

Non-contrast Cardiac CT Result -- Part 1 [sct_Noncontrast_Result1]

# ✔	Item Number	Non-Contrast CT Catogeries	Result (integer)	Units
3.a	1	Global Calcium score		N/A
3.b	2	Left Main calcium score		N/A
3.c	3	Interventricular artery calcium score		N/A
3.d	4	Circumflex artery calcium score		N/A
3.e	5	Right coronary artery calcium score		N/A

Non-contrast Cardiac CT Result -- Part 1 Entry [sct_Noncontrast_Result1]

3.1	Item Number<br>[Item Number]	**[itm_Item_Number]**<br>**[cod_Row]**
3.2	Non-Contrast CT Catogeries<br>[Non-Contrast CT Catogeries]	**[itm_NonContrastCT_Cat]**<br>**[cod_NonContrast_Cat]**
3.3	Result (integer)<br>[Result (integer)]	**[itm_Sub_Result_Integer]**
3.4	Units<br>[Units]	**[itm_LabUnit]**<br>**[cod_Units]**

Non-contrast Cardiac CT Result -- Part 2 [sct_NonContrast_Result2]

# ✔	Item Number	Non-Contrast CT Catogeries	Result (2 decimal places)	Units
4.a	6	Age and Sex calcium score percentile		%
4.b	7	Epicardial Adipose volume		cm3

Non-contrast Cardiac CT Result -- Part 2 Entry [sct_NonContrast_Result2]

4.1	Item Number<br>[Item Number]	**[itm_Item_Number]**<br>**[cod_Row]**
4.2	Non-Contrast CT Catogeries<br>[Non-Contrast CT Catogeries]	**[itm_NonContrastCT_Cat]**<br>**[cod_NonContrast_Cat]**
4.3	Result (2 decimal places)<br>[Result (2 decimal places)]	**[itm_Sub_Result_Float]**
4.4	Units<br>[Units]	**[itm_LabUnit]**<br>**[cod_Units]**

The Canadian HIV and Aging Cohort - Determinanats of increased risk of cardio-vascular diseases in HIV-infected individuals: Sub-study -- Oral Glucose Tolerance Test (Sub-study -- OGTT) [frm_Sub_OGTT]

Sub-study -- Oral Glucose Tolerance Test [frm_Sub_OGTT]

1.	Date patient had exam<br>[Date patient had exam]	**[itm_ExamDate]**<br>/ /
2.	Blood Sample Frozen for Research<br>[Blood Sample Frozen for Research]	**[itm_Blood_Frozen]**<br>Yes No

Oral Glucose Tolerance Test Result [sct_OGT]

# ✔	Item Number	OGT Catogeries	Result (2 decimal places)	Units
3.a	1	Fasting Glycemia		mmol/L
3.b	2	Glycemia at 30 minutes		mmol/L
3.c	3	Glycemia at 1 hour		mmol/L
3.d	4	Glycemia at 2 hours		mmol/L

Oral Glucose Tolerance Test Result Entry [sct_OGT]

3.1	Item Number<br>[Item Number]	**[itm_Item_Number]**<br>**[cod_Row]**
3.2	Oral Glucose Tolerance Test Catogeries<br>[OGT Catogeries]	**[itm_OGT_Cat]**<br>Fasting Glycemia Glycemia at 30 minutes Glycemia at 1 hour Glycemia at 2 hours
3.3	Result (2 decimal places)<br>[Result (2 decimal places)]	**[itm_Sub_Result_Float]**
3.4	Units<br>[Units]	**[itm_LabUnit]**<br>**[cod_Units]**

The Canadian HIV and Aging Cohort - Determinanats of increased risk of cardio-vascular diseases in HIV-infected individuals: Sub-study -- Cardiac TEP Scan (Sub-study -- Cardiac TEP Scan) [frm_Sub_TEP]

Sub-study -- Cardiac TEP Scan [frm_Sub_TEP]

1.	Date patient had exam<br>[Date patient had exam]	**[itm_ExamDate]**<br>/ /
2.	Able to perform exam (technically impossible)<br>[Able to perform exam (technically impossible)]	**[itm_ExamAble]**<br>○Yes ○No

TEP Scan Result [sct_TEP_Result]

# ✔	Item Number	TEP Scan Categories	Result (2 decimal places)
3.a	1	Carotid maximum TBR ratio	
3.b	2	Carotid mean TBR ratio	
3.c	3	Ascending Aorta maximum TBR ratio	
3.d	4	Ascending Aorta mean TBR ratio	

TEP Scan Result Entry [sct_TEP_Result]

3.1	Item Number<br>[Item Number]	**[itm_Item_Number]**<br>**[cod_Row]**
3.2	TEP Scan Categories<br>[TEP Scan Categories]	**[itm_TEP_Cat]**<br>○Carotid maximum TBR ratio ○Carotid mean TBR ratio ○Ascending Aorta maximum TBR ratio ○Ascending Aorta mean TBR ratio
3.3	Result (2 decimal places)<br>[Result (2 decimal places)]	**[itm_Sub_Result_Float]**

**The Canadian HIV and Aging Cohort - Determinanats of increased risk of cardio-vascular diseases in HIV-infected individuals: Metabolic Syndrome Criteria Present (Metabolic Syndrome Criteria Present) [frm_MetS_Criteria]**		
**Metabolic Syndrome Criteria Present [frm_MetS_Criteria]**		
1.	Criteria Present?<br>[Criteria Present?]	**[itm_CriteriaPresent]**<br>◯Yes ◯No

The Canadian HIV and Aging Cohort - Determinanats of increased risk of cardio-vascular diseases in HIV-infected individuals: Metabolic Syndrome (Metabolic Syndrome) [frm_MetS]

Metabolic Syndrome [frm_MetS]

1.	Yes<br>Waist Circumference >94 cm men or >80 cm women?<br>[Waist Circumference]	**[itm_MetS_Waist]**<br>Yes<br>No
2.	Triglycerides >1.7 mmol/L?<br>[Triglycerides >1.7 mmol/L]	**[itm_MetS_TG_Value]**<br>Yes<br>**[itm_MetS_TG_Tx]**<br>No<br>Treatment for raised triglycerides?<br>Yes<br>No
3.	HDL <0.9 mmol/L for men or <1.1 mmol/L in woman?<br>[HDL over?]	**[itm_MetS_HDL]**<br>Yes<br>No
4.	Systolic blood pressure >130 mmHg or Diastolic blood pressure > 85 mmHg?<br>[BP is higher than the normal range]	**[itm_MetS_BP_Value]**<br>Yes<br>**[itm_MetS_BP_Tx]**<br>No<br>Treatment for hypertension?<br>Yes<br>No
5.	Elevated fasting plasma glucose > 5.6 mmol/L?<br>[Elevated fasting plasma glucose > 5.6 mmol/L?]	**[itm_MetS_Glu_Value]**<br>Yes<br>**[itm_MetS_Glu_Tx]**<br>No<br>Drug treatment for elevated glucose?<br>Yes<br>No
6.	Is Metabolic Syndrome present?<br>*(Metabolic syndrome present if criteria #1 and at least 2/4 of criteria 2-5 are present.)*<br>*[Is Metabolic Syndrome present?]*	**[itm_MetS_Present]**<br>Yes No

The Canadian HIV and Aging Cohort - Determinanats of increased risk of cardio-vascular diseases in HIV-infected individuals: Diabetes Diagnosis (DM2 Diagnosis) [frm_DMDiag]

Diabetes Diagnosis [frm_DMDiag]

1.	Criteria Present?<br>[Criteria Present?]	**[itm_CriteriaPresent]**<br>○ **[itm_DMpresent]**<br>Is Diabetes present?<br>*(At least one the 4 criterias must be met on 2 different days)*<br>○ Yes<br>○ No<br>○ No

The Canadian HIV and Aging Cohort - Determinanats of increased risk of cardio-vascular diseases in HIV-infected individuals: Diabetes Type 2 -- First Evaluation (DM2_1st) [frm_DMDiag_1st]

Diabetes Type 2 -- First Evaluation [frm_DMDiag_1st]

1.	Diabetes Criteria Date<br>[Diabetes Criteria Date]	**[itm_DMCriteriaDate]**<br>/ /

Diabetes Type 2 -- First Criteria Met [sct_DM2Criteria_1st]

Symptoms = polyuria, polydipsia or unexplained weight loss

# ✔	Diabetes Criteria Met Evaluation	Diabetes Criteria	Diabetes Criterion Met?
2.a	1	Fasting plasma glucose > 7.0 mmol/L?	
2.b	1	Casual plasma glucose > 11.0 mmol/L with symptoms of diabetes?	
2.c	1	2 h plasma glucose in 75 g glucose tolerance test > 11.1 mmol/L?	
2.d	1	HbA1c > 6 %	

Diabetes Type 2 -- First Criteria Met Entry [sct_DM2Criteria_1st]

Symptoms = polyuria, polydipsia or unexplained weight loss

2.1	Diabetes Criteria Met Evaluation<br>[Diabetes Criteria Met Evaluation]	**[itm_DMCriteriaMet_Ev]**<br>**[cod_Row]**
2.2	Diabetes Criteria<br>[Diabetes Criteria]	**[itm_DMCriteria]**<br>○ Fasting plasma glucose > 7.0 mmol/L? ○ Casual plasma glucose > 11.0 mmol/L with symptoms of diabetes? ○ 2 h plasma glucose in 75 g glucose tolerance test > 11.1 mmol/L? ○ HbA1c > 6 %
2.3	Diabetes Criterion Met?<br>[Diabetes Criterion Met?]	**[itm_DMCriterionMet]**<br>○ Yes ○ No

The Canadian HIV and Aging Cohort - Determinanats of increased risk of cardio-vascular diseases in HIV-infected individuals: Diabetes Diagnosis -- 2nd Evaluation (DM2_2nd) [frm_DMDiag_2nd]

Diabetes Diagnosis -- 2nd Evaluation [frm_DMDiag_2nd]

1.	Diabetes Criteria Date<br>[Diabetes Criteria Date]	**[itm_DMCriteriaDate]**<br>/ /

Diabetes Type 2 -- Second Criteria Met [sct_DM2Criteria_2nd]

(Symptoms = polyuria, polydipsia or unexplained weight loss)

# ✔	Diabetes Criteria Met Evaluation	Diabetes Criteria	Diabetes Criterion Met?
2.a	2	Fasting plasma glucose > 7.0 mmol/L?	
2.b	2	Casual plasma glucose > 11.0 mmol/L with symptoms of diabetes?	
2.c	2	2 h plasma glucose in 75 g glucose tolerance test > 11.1 mmol/L?	
2.d	2	HbA1c > 6 %	

Diabetes Type 2 -- Second Criteria Met Entry [sct_DM2Criteria_2nd]

(Symptoms = polyuria, polydipsia or unexplained weight loss)

2.1	Diabetes Criteria Met Evaluation<br>[Diabetes Criteria Met Evaluation]	**[itm_DMCriteriaMet_Ev]**<br>**[cod_Row]**
2.2	Diabetes Criteria<br>[Diabetes Criteria]	**[itm_DMCriteria]**<br>○ Fasting plasma glucose > 7.0 mmol/L? ○ Casual plasma glucose > 11.0 mmol/L with symptoms of diabetes? ○ 2 h plasma glucose in 75 g glucose tolerance test > 11.1 mmol/L? ○ HbA1c > 6 %
2.3	Diabetes Criterion Met?<br>[Diabetes Criterion Met?]	**[itm_DMCriterionMet]**<br>○ Yes ○ No

The Canadian HIV and Aging Cohort - Determinanats of increased risk of cardio-vascular diseases in HIV-infected individuals: Quality of Life Questionnaire Language Choice (QOL Language Choice) [frm_QOL_Language]		
**Quality of Life Questionnaire Language Choice [frm_QOL_Language]**		
1.	What language is used?<br>[Language?]	**[itm_QOL_Language]**<br>English French

The Canadian HIV and Aging Cohort - Determinanats of increased risk of cardio-vascular diseases in HIV-infected individuals: Quality of life Questionnaire (QOL) [frm_QOL]

Quality of life questionnaire [sct_QOL]

# ✔	Item Number	QOL Item	QOL Frequency
1.a	1	You felt depressed	
1.b	2	You felt anxious	
1.c	3	You felt needed	
1.d	4	You worried about many things	
1.e	5	You had health problems	
1.f	6	You were too tired to do the things you wanted to do	
1.g	7	You were bothered by nausea	
1.h	8	You were bothered by physical discomfort	
1.i	9	You were able to do things around the house	
1.j	10	You were able to get from place to place (e.g., to shop, to go to the doctor)	
1.k	11	You were able to climb several flights of stairs without help	
1.l	12	You could perform strenuous sports like running or weight training	
1.m	13	You felt isolated	
1.n	14	You withdrew from socializing	
1.o	15	You lacked energy to socialize with friends	
1.p	16	You spent quality time with friends	
1.q	17	You received enough emotional support from people close to you	
1.r	18	You had someone who could help you in case of an emergency	
1.s	19	You had someone you could talk to about your problems	
1.t	20	You had someone who could give you useful advice	
1.u	21	You were bothered by trouble remembering things	
1.v	22	You were bothered by forgetting what you started to do	
1.w	23	You found that your mind wandered more than usual	
1.x	24	You were bothered by a short attention span	
1.y	25	You had to put off paying regular living expenses	
1.z	26	You did not have enough money to pay for medication ***(If you had to pay for medication, would you have been short on money?)***	
1.aa	27	You did not have enough money to pay for recreational activities	
1.ab	28	You were concerned about your financial future	
1.ac	29	You felt satisfied with your activities with your spouse or your partner	
1.ad	30	You felt satisfied with the amount of affection your spouse or partner expressed toward you	
1.ae	31	You were able to confide in your spouse or partner	
1.af	32	You felt mistrust for your spouse or partner	
1.ag	33	You felt satisfied with your sexual activity (sex life) ***(Were you satisfied with not having any sexual activity?)***	
1.ah	34	You wanted sex more often	
1.ai	35	You were afraid of infecting someone through sexual contact	
1.aj	36	You were afraid of being rejected in a sexual context	
1.ak	37	You wanted more information from your health care providers	
1.al	38	You wanted more support from your doctors and other health providers	
1.am	39	You doubted that your health care providers were doing all they could for your health	
1.an	40	You were able to get the medical care you needed	

Quality of life questionnaire Entry [sct_QOL]

1.1	Item Number<br>[Item Number]	**[itm_QOLItem_Number]**<br>**[cod_Row]**
1.2	In the past two weeks, with what frequency did you feel or experience the following things?<br>[QOL Item]	**[itm_QOLItem]**<br>**[cod_QOLItem]**
1.3	Choose one of the following answers:<br>[QOL Frequency]	**[itm_QOL_Freq]**<br>○ 1 (Never) ○ 2 (Very rarely) ○ 3 (Sometimes) ○ 4 (Half of the time) ○ 5 (Quite often) ○ 6 (Very often) ○ 7 (Always) ○ 8 (Not applicable)

The Canadian HIV and Aging Cohort - Determinanats of increased risk of cardio-vascular diseases in HIV-infected individuals: Questionnaire sur votre état de santé et votre qualité de vie (QOL -- French) [frm_QOL_French]

Questionnaire sur votre état de santé et votre qualité de vie [sct_QOL_French]

# ✔	Item Number	QOL Item	QOL Frequency
1.a	1	Vous vous sentiez déprimé(e)	
1.b	2	Vous vous sentiez anxieux(se)	
1.c	3	Vous sentiez qu'on avait besoin de vous	
1.d	4	Vous vous inquiétiez de plusieurs choses	
1.e	5	Vous aviez des problèmes de santé	
1.f	6	Vous étiez trop fatigué(e) pour faire les choses que vous vouliez faire	
1.g	7	Vous étiez incommodé(e) par des nausées	
1.h	8	Vous étiez incommodé e) par des malaises physiques	
1.i	9	Vous étiez capable de faire des tâches ou travaux de maison (travaux domestiques)	
1.j	10	Vous étiez capable de vous déplacer d'un endroit à l'autre (ex. aller magasiner ou aller chez le médecin)	
1.k	11	Vous étiez capable de monter plusieurs paliers d'escaliers sans aide	
1.l	12	Vous pouviez faire des activités sportives exigeantes (courir, faire de la musculation, etc.)	
1.m	13	Vous vous sentiez isolé(e)	
1.n	14	Vous vous êtes retiré(e) de votre vie sociale	
1.o	15	avez manqué d'énergie pour socialiser avec des ami(e)s	
1.p	16	Vous avez passé du temps privilégié avec des ami(e)s	
1.q	17	Vous avez reçu assez de soutien émotif des gens proches de vous	
1.r	18	Vous aviez quelqu'un qui pouvait vous aider en cas d'urgence	
1.s	19	Vous aviez quelqu'un à qui vous pouviez parler de vos problèmes	
1.t	20	Vous aviez quelqu'un qui pouvait vous donner des conseils utiles	
1.u	21	Vous avez été dérangé(e) par des troubles de mémoire	
1.v	22	Vous avez été dérangé(e) par le fait d'oublier ce que vous commenciez à faire	
1.w	23	Vous avez été distrait(e) plus que d'habitude	
1.x	24	Vous avez été dérangée(e) par une capacité limitée de concentration	
1.y	25	Vous avez dû remettre à plus tard le paiement de dépenses courantes	
1.z	26	Vous avez manqué d'argent pour payer des médicaments ***(Si vous aviez eu à acheter des médicaments, auriez-vous manqué d'argent?)***	
1.aa	27	Vous avez manqué d'argent pour vous payer des loisirs	
1.ab	28	Vous étiez inquiet(ète) à propos de votre avenir financier	
1.ac	29	Vous étiez satisfait(e) de vos activités avec votre conjoint(e) ou partenaire	
1.ad	30	Vous étiez satisfait(e) du niveau d'affection que vous a exprimé votre conjoint(e) ou partenaire	
1.ae	31	Vous pouviez faire des confidences à votre conjoint(e) ou partenaire	
1.af	32	Vous ressentiez de la méfiance envers votre conjoint(e) ou partenaire	
1.ag	33	Vous étiez satisfait(e) de vos activités sexuelles (vie sexuelle) ***(Étiez-vous satisfait de ne pas avoir eu d'activités sexuelles?)***	
1.ah	34	Vous auriez voulu des relations sexuelles plus souvent	
1.ai	35	Vous avez eu peur d'infecter quelqu'un par contact sexuel	
1.aj	36	Vous avez eu peur d'être rejeté(e) sexuellement	
1.ak	37	Vous vouliez plus d'information de la part de ceux qui vous fournissent des soins de santé	
1.al	38	Vous vouliez plus de soutien de la part de vos médecins et de ceux qui vous fournissent des soins de santé	
1.am	39	Vous aviez des doutes quant au fait que ceux qui vous fournissent des soins de santé faisaient tout ce qu'ils pouvaient pour votre santé	
1.an	40	Vous pouviez obtenir les soins médicaux dont vous aviez besoin	

Questionnaire sur votre état de santé et votre qualité de vie Entry [sct_QOL_French]

1.1	Numéro de repère<br>[Item Number]	**[itm_QOLItem_Number_French]**<br>**[cod_Row]**
1.2	Au cours des deux dernières semaines, à quelle fréquence avez-vous ressenti ou fait les choses suivantes?<br>[QOL Item]	**[itm_QOLItem_French]**<br>**[cod_QOLItem_French]**
1.3	Répondez par:<br>[QOL Frequency]	**[itm_QOL_Freq_French]**<br>○ 1 (Jamais) ○ 2 (Très rarement) ○ 3 (Quelquefois) ○ 4 (La moitié du temps) ○ 5 (Assez souvent) ○ 6 (Très souvent) ○ 7 (Tout le temps) ○ 8 (Ne s'applique pas)

The Canadian HIV and Aging Cohort - Determinanats of increased risk of cardio-vascular diseases in HIV-infected individuals: Antiretrovirals Medication (ARV Medication) - Repeating Form [frm_ARVMed]

#	Medication No,	What ARV treatment the patient has/had?	Drug Name (Generic or proprietary)	Dose (e.g. 10 mg)	Frequency (e.g. BID)	Start Date	Ongoing at the end of the study?
1							

Antiretrovirals Medication [frm_ARVMed]

1.	Medication No.<br>[Medication No,]	**[itm_Row]**
2.	What ARV treatment the patient has/had?<br>[What ARV treatment the patient has/had?]	**[itm_ARVTxType_French]**<br>**[cod_ARVTxType]**
3.	Drug Name (Generic or proprietary)<br>[Drug Name (Generic or proprietary)]	**[itm_DrugName]**
4.	Dose (e.g. 10 mg)<br>[Dose (e.g. 10 mg)]	**[itm_Dose]**
5.	Frequency (e.g. BID)<br>[Frequency (e.g. BID)]	**[itm_Frequency]**
6.	Start Date<br>[Start Date]	**[itm_StartDate]**<br>/ /
7.	Ongoing at the end of the study?<br>[Ongoing at the end of the study?]	**[itm_OngoingEndStudy]**<br>Yes<br>**[itm_StopDate]**<br>No<br>Stop Date<br>/ /

The Canadian HIV and Aging Cohort - Determinanats of increased risk of cardio-vascular diseases in HIV-infected individuals: Concomitant Medications (Concomitant Medications) - Repeating Form [frm_ConMed]

#	Medication No,	Drug Name (Generic or proprietary)	Dose (e.g. 10 mg)	Frequency (e.g. BID)	Indication	Start Date	Ongoing at the end of the study?
1							

Concomitant Medications [frm_ConMed]

1.	Medication No.<br>[Medication No,]	**[itm_Row]**
2.	Drug Name (Generic or proprietary)<br>[Drug Name (Generic or proprietary)]	**[itm_DrugName]**
3.	Dose (e.g. 10 mg)<br>[Dose (e.g. 10 mg)]	**[itm_Dose]**
4.	Frequency (e.g. BID)<br>[Frequency (e.g. BID)]	**[itm_Frequency]**
5.	Indication<br>[Indication]	**[itm_Indiccation]**
6.	Start Date<br>[Start Date]	**[itm_StartDate]**<br>/ /
7.	Ongoing at the end of the study?<br>[Ongoing at the end of the study?]	**[itm_OngoingEndStudy]**<br>○ Yes<br>○ **[itm_StopDate]**<br>No<br>Stop Date<br>/ /

The Canadian HIV and Aging Cohort - Determinanats of increased risk of cardio-vascular diseases in HIV-infected individuals: Medical Event -- CVD (ME -- CVD) [frm_ME_CVD]

Please only include CVD related medical events.

	Row	CVD related disease or procedure?	Specify	Onset Date	Therapy for the Event?	Disease Status
1. ✓						

CVD Disease Entry [sct_CVDDisPro]

1.1	Row<br>[Row]	**[itm_RowMH]**
1.2	Is it a CVD related disease or procedure?<br>[CVD related disease or procedure?]	**[itm_CVD_DisPro]**<br>◯ CVD Related Disease ◯ CVD Related Procedure
1.3	Specify<br>[Specify]	**[itm_Disease]**
1.4	Onset Date<br>[Onset Date]	**[itm_OnSetDate]**<br>/ /
1.5	Therapy for the Event?<br>[Therapy for the Event?]	**[itm_Therapy]**<br>◯ Yes ◯ No ◯ Irretrievable
1.6	Disease Status<br>[Disease Status]	**[itm_DiseaseStatus]**<br>◯ 1: Active stable<br>◯ 2: Active unstable<br>◯ **[itm_ResolveDate]**<br>3: Resolved<br>Resolution Date<br>/ /<br>◯ 4: Unknown<br>◯ Irretrievable
1.7	Modified Term<br>[Modified Term]	**[itm_ModifiedTerm]**
1.8	MedDRA synonym<br>[MedDRA synonym]	**[itm_MedDRA]**
1.9	Failed Coding<br>[Failed Coding]	**[itm_FailedCoding]**
1.10	System Organ Class<br>[SOC]	**[itm_SOC]**
1.11	High Level Group Term<br>[HLGT]	**[itm_HLGT]**
1.12	High Level Term<br>[HLT]	**[itm_HLT]**
1.13	Prefered Term<br>[PT]	**[itm_PT]**
1.14	Low Lever Term<br>[LLT]	**[itm_LLT]**
1.15	Low Lever Term Code<br>[LLT Code]	**[itm_LLTCODE]**
1.16	AE English (Hidden)<br>[AE English (Hidden)]	**[itm_AEHiddenE]**

The Canadian HIV and Aging Cohort - Determinanats of increased risk of cardio-vascular diseases in HIV-infected indivi

	Row	Organ, System or Disease	
1. ✓			

Medical Event -- HIV Entry [sct_ME_HIV]

1.1	Row [Row]	**[itm_RowMH]**
1.2	Organ, System or Disease [Organ, System or Disease]	**[itm_OrganClass_HIV]** **[codOrganSystemDisease_HIV_1]**
1.3	HIV or AIDS Defining Conditions? [HIV or AIDS Defining Conditions?]	**[itm_Rel_HIVAIDS]**<br>◯ **[itm_EventCode_HIV]** HIV Defining Conditions (01-15) Event Code<br>☐ 01= Seborrheic dermatitis ☐ 02= Persistent generalized lymphadenopathy >1month ☐ 03= Fever higher than 38.5 C or 101.3 F Otherwise undisgnosed ☐ 04= Idiopathic thrombocytopenic purpura (ITP) ☐ 05= ((cand<br>◯ **[itm_EventCode_AIDS]** AIDS Defining Conditions (16-40) Event Code<br>☐ 16= Candidiasis of bronchi, trachea or lungs ☐ 17= Candidiasis, esophageal ☐ 18= Cervical cancer invasive ☐ 19= Coccidioidomycosis, disseminated or extrapulmonary ☐ 20= Cryptococcosis, extrapulmonary ☐ 21= Cryptosporidiosis, chronic intestinal (greater than 1 month's duration) ☐ 22= Cytomegalovirus disease (other than liver, spleen or nodes) ☐ 23= Cytomegalovirus retinitis (with loss of vision) ☐ 2 E H
1.4	Specify [Specify]	**[itm_Disease]**
1.5	Onset Date [Onset Date]	**[itm_OnSetDate]**<br>/ /
1.6	Therapy for the Event? [Therapy for the Event?]	**[itm_Therapy]**<br>◯ Yes ◯ No ◯ Irretrievable
1.7	Disease Status [Disease Status]	**[itm_DiseaseStatus]**<br>◯ 1: Active stable<br>◯ 2: Active unstable<br>◯ **[itm_ResolveDate]** 3: Resolved Resolution Date / /<br>◯ 4: Unknown<br>◯ Irretrievable
1.8	Modified Term [Modified Term]	**[itm_ModifiedTerm]**
1.9	MedDRA synonym [MedDRA synonym]	**[itm_MedDRA]**
1.10	Failed Coding [Failed Coding]	**[itm_FailedCoding]**
1.11	System Organ Class [SOC]	**[itm_SOC]**
1.12	High Level Group Term [HLGT]	**[itm_HLGT]**
1.13	High Level Term [HLT]	**[itm_HLT]**
1.14	Prefered Term [PT]	**[itm_PT]**
1.15	Low Lever	**[itm_LLT]**

	Term [LLT]	
1.16	Low Lever Term Code [LLT Code]	[itm_LLTCODE]
1.17	AE English (Hidden) [AE English (Hidden)]	[itm_AEHiddenE]

The Canadian HIV and Aging Cohort - Determinanats of increased risk of cardio-vascular diseases in HIV-infected individuals: Medical Events -- Non-HIV NonCVD (ME -- Others) [frm_ME_nonHIVCVD]

	Row	SOC	Specify	Onset Date	Therapy for the Event?	Disease Status
1. ✓						

Medical Event Entry [sct_ME_nonHIV]

1.1	Row<br>[Row]	**[itm_RowMH]**
1.2	System Organ Class<br>[SOC]	**[itm_OrganClass]**<br>**[cod_OrganClass]**
1.3	Specify<br>[Specify]	**[itm_Disease]**
1.4	Onset Date<br>[Onset Date]	**[itm_OnSetDate]**<br>/ /
1.5	Therapy for the Event?<br>[Therapy for the Event?]	**[itm_Therapy]**<br>○ Yes ○ No ○ Irretrievable
1.6	Disease Status<br>[Disease Status]	**[itm_DiseaseStatus]**<br>○ 1: Active stable<br>○ 2: Active unstable<br>○ **[itm_ResolveDate]**<br>3: Resolved<br>Resolution Date<br>/ /<br>○ 4: Unknown<br>○ Irretrievable
1.7	Modified Term<br>[Modified Term]	**[itm_ModifiedTerm]**
1.8	MedDRA synonym<br>[MedDRA synonym]	**[itm_MedDRA]**
1.9	Failed Coding<br>[Failed Coding]	**[itm_FailedCoding]**
1.10	System Organ Class<br>[SOC]	**[itm_SOC]**
1.11	High Level Group Term<br>[HLGT]	**[itm_HLGT]**
1.12	High Level Term<br>[HLT]	**[itm_HLT]**
1.13	Prefered Term<br>[PT]	**[itm_PT]**
1.14	Low Lever Term<br>[LLT]	**[itm_LLT]**
1.15	Low Lever Term Code<br>[LLT Code]	**[itm_LLTCODE]**
1.16	AE English (Hidden)<br>[AE English (Hidden)]	**[itm_AEHiddenE]**

The Canadian HIV and Aging Cohort - Determinanats of increased risk of cardio-vascular diseases in HIV-infected individuals: Hospitalizations (Hospitalizations) [frm_Hospt]

	Row	Diagnosis of Admission	Admission Date	Admission Date	Discharged To
1. ✔					

Hospitalizations Entry [sct_Hospitalizations]

1.1	Row<br>[Row]	**[itm_Hosp_Row]**
1.2	Diagnosis of Admission<br>[Diagnosis of Admission]	**[itm_Hosp_Diag]**
1.3	Admission Date<br>[Admission Date]	**[itm_Hosp_AdmiDate]**<br>/ /
1.4	Discharge Date<br>[Admission Date]	**[itm_Hosp_Disc_Date]**<br>/ /
1.5	Discharged To<br>[Discharged To]	**[itm_DischargedTo]**<br>○ Home ○ Other Institution ○ Death
1.6	Modified Term<br>[Modified Term]	**[itm_ModifiedTerm]**
1.7	MedDRA synonym<br>[MedDRA synonym]	**[itm_MedDRA]**
1.8	Failed Coding<br>[Failed Coding]	**[itm_FailedCoding]**
1.9	System Organ Class<br>[SOC]	**[itm_SOC]**
1.10	High Level Group Term<br>[HLGT]	**[itm_HLGT]**
1.11	High Level Term<br>[HLT]	**[itm_HLT]**
1.12	Prefered Term<br>[PT]	**[itm_PT]**
1.13	Low Lever Term<br>[LLT]	**[itm_LLT]**
1.14	Low Lever Term Code<br>[LLT Code]	**[itm_LLTCODE]**

The Canadian HIV and Aging Cohort - Determinanats of increased risk of cardio-vascular diseases in HIV-infected individuals: Biobank-frozen Biological Samples (Biobank-frozen Biological Samples) [frm_Biobank]

Biobank-frozen Biological Samples [frm_Biobank]

1.	Total -- PBMC<br>[Total PBMC]	**[itm_BiobankTotal_PBMC]**
2.	Total -- Plasma<br>[Total Plasma]	**[itmBiobankTotal_Plasma]**
3.	Total -- Serum<br>[Total Serum]	**[itmBiobankTotal_Serum]**

	Blood Processed Date	Entry or withdrawal?	Name of Researcher	Types of biological samples	Amount	Unit
4. ✔						

Biobank-frozen Biological Samples Entry [sct_Biobank]

4.1	Blood Processed Date<br>[Blood Processed Date]	**[itm_ProcessedDate]**<br>/ /
4.2	Entry or withdrawal from the Bio-bank?<br>[Entry or withdrawal?]	**[itm_Biobank_EntryWithdl]**<br>○ Entry ○ Withdrawal
4.3	Name of Researcher<br>[Name of Researcher]	**[itm_Biobank_Researcher]**
4.4	Types of biological samples<br>[Types of biological samples]	**[itm_Biobank_Types]**<br>○ PBMC ○ Plasma ○ Serum
4.5	Amount<br>[Amount]	**[itm_Biobank_Amount]**
4.6	Unit<br>[Unit]	**[itm_Biobank_Unit]**<br>○ xE6/L ○ Vial

The Canadian HIV and Aging Cohort - Determinanats of increased risk of cardio-vascular diseases in HIV-infected individuals: Unscheduled Reason (Unscheduled Reason) [frm_UnschReason]		
**Reasons [sct_Reasons]**		
1.	Reason for this Unscheduled Visit<br>[Reason for this Unscheduled Visit]	**[itm_Reason_MissedVisit]**
**Forms Completed in this unscheduled visit [sct_Forms]**		
2.	CVD Risks (Smoking, Alcohol and Drug Abuse)<br>[CVD Risks]	**[itm_Form_CVDRisks]**<br>Yes
3.	Physical Exam<br>[Physical Exam]	**[itm_Form_PE]**<br>Yes
4.	Vital Signs<br>[Vital Signs]	**[itm_Form_VS]**<br>Yes
5.	Metabolic Syndrome<br>[Metabolic Syndrome]	**[itm_Form_MetS]**<br>Yes
6.	Type 2 Diabetes<br>[Type 2 Diabetes]	**[itm_Form_Type2DM]**<br>Yes
7.	Bone Mineral Density<br>[Bone Mineral Density]	**[itm_Form_BMD]**<br>Yes
8.	Laboratory<br>[Laboratory]	**[itm_Form_Lab]**<br>Yes
9.	Other Tests<br>[Other Tests]	**[itm_Form_OtherTests]**<br>Yes

The Canadian HIV and Aging Cohort - Determinanats of increased risk of cardio-vascular diseases in HIV-infected individuals: Study Completion (Study Completion) [frm_StudyComplt]		
**Study Completion [frm_StudyComplt]**		
1.	Date of subject last visit date<br>[Last visit date]	**[itm_LastDate]**<br>/ /
2.	Subject completed study?<br>[Subject completed study?]	**[itm_Complt]**<br>Yes<br>**[itm_DropRea]**<br>No<br>Reason for subject going off-study (select all that applies)<br>Adverse Event(s)<br>Protocol Violation<br>Consent withdrew<br>Efficacy<br>Lost to follow up<br>Study stopped<br>Death<br>**[itm_Spec_Other]**<br>Other<br>Specify

The Canadian HIV and Aging Cohort - Determinanats of increased risk of cardio-vascular diseases in HIV-infected individuals: Study Completion -- Death (Study Completion -- Death) [frm_StudyComplt_Death]

Study Completion -- Death [frm_StudyComplt_Death]

1.	Death<br>Date of Death<br>[Date of Death]	**[itm_DeathDate]**<br>/ /
2.	Cause of death<br>[Cause of death]	**[itm_DeathCause]**<br>Cardiovascular<br>**[itm_DeathCause_Spec_Other]**<br>Other<br>Specify
3.	Was autopsy performed?<br>[Was autopsy performed?]	**[itm_Autopsy]**<br>**[itm_Results]**<br>Yes<br>Results<br>No
