# Supplementary material for: The Canadian HIV and aging cohort study - determinants of increased risk of cardio-vascular diseases in HIV-infected individuals: rationale and study protocol
Source: BMC Infect Dis. 2017 Sep 11;17:611. doi: 10.1186/s12879-017-2692-2 (PMC5594495; doi:10.1186/s12879-017-2692-2)
Supplement: Supplementary file 5 — Supplementary Statistical Analysis for sub studies. (DOCX 24 kb) [file 12879_2017_2692_MOESM5_ESM.docx]

**Additional File 5 – Supplementary Statistical Analysis for sub studies**

| **Details of planned statistical analysis for Sub-Studies** | |
| --- | --- |
| **1 – Characterization of atherosclerotic plaque** | **Positron emitting tomography (PET) scan of the ascending aorta and carotid arteries:** For each participant, mean target-to-background ratio for both carotids and ascending aorta will be calculated. The co-primary outcome will be difference in mean target-to-background ratio in both carotids and in ascending aorta between HIV participants and HIV negative controls. This will be assessed using the Wilcoxon signed rank test. The secondary endpoint will be calculated similarly, using the maximal target-to-background ratio as measure of interest.  **Carotid arteries ultrasound with measurement of intima-media thickness:** For each subject, the yearly rate of change in c-IMT will be estimated through simple linear regression fit to 3 c-IMT values (baseline, 2 and 4 years).The primary outcome will be the differences in the mean yearly rates of C-IMT increase between the HIV+ and HIV, as measured with a T-test.  **Cardiac computed tomography scan without injection of contrast media:** Calcium scores will be determined at baseline. The Agatston–Janowitz calcium score will be expressed as age- and sex-adjusted percentiles. For each subject, rate of change in calcium score will be estimated as the difference in calcium score values at 4 years – baseline, divided by 4 years.  The primary outcome will be increase in calcium score (estimated as the difference between values at 4 years versus baseline, divided by time difference). If necessary, the original values of progression rates will be transformed, using e.g. logarithmic transformation, to ensure normality of their distributions. The independent effect of HIV status will be estimated through the adjusted mean difference in the progression rates of HIV+ versus HIV- negative subjects, with 95% CI, and its significance will be tested with the model-based F-test at α=0.05.  For the subset of participants who will undergo both CAC and C-IMT, correlations between CAC and C-IMT will be obtained using mixed linear models, both for baseline values and for progression rates.  **Cardiac computed tomography scan with injection of contrast media:** The primary outcome will be difference in total plaque volume according to HIV status, after adjustment for cardiovascular risk factors, using linear regression analysis. For participants undergoing cardiac computed tomography with and without contrast media, the correlation between the presence of atherosclerosis (coded as binary) on both exams will be assessed according to HIV infection status, using mixed model logistic regression with an interaction term for HIV infection. Differences in non-calcified plaque volume and calcified plaque volume, adjusted for cardiovascular risk factors, will be modelled using multivariable linear regression. Coronary artery stenosis will be described and the number of lesions >50% will be compared using the Wilcoxon signed-rank test.  **Intravascular coronary ultrasound:** The primary outcome will be comparison of plaque characteristics (defined as percentages of fibrous, fibro-fatty, necrotic core or dense-calcium plaque tissue) between HIV-infected and HIV-uninfected participant, modeled using linear mixed models. Secondary outcomes will be the proportion of each plaque type, in HIV-infected vs. uninfected, modeled using mixed logistic regression models. |
| **2 – Characterization of dysglycemia** | Incidence rates for dysglycaemia and the MetS will be calculation using survival analysis models. The response to the OGTT of the plasma glucose, insulin, and the markers of oxidative stress, inflammation, coagulation and endothelial dysfunction will be calculated as the incremental area under the curve (AUC). These will be compared between the two groups according to HIV status using Student T-test. For participants who also underwent CAC, contrast-enhanced CT and C-IMT, the AUC of those parameters will also be correlated to C-IMT, CAC scores and coronary artery plaque volume using logistic regression analysis. |
| **3 – Characterization of immune profiles in CVD** | The reference group for all analysis will be the HIV-negative participants.  ***TH17 paucity:*** The co-primary outcome will be difference in levels of peripheral blood Th17 cells between HIV-positive participants with CVD and HIV negative, and between HIV-positive participants without CVD and HIV negative. This will be modeled using multiple linear regression, with adjustment for baseline risk factors of CVD.  ***Monocyte activation:*** The primary outcome will be the extent of peripheral blood monocyte activation between HIV-positive participants with CVD and HIV negative with CVD, and between HIV-positive participants without CVD and HIV negative without CVD. This will be modeled using multiple linear regression, with adjustment for baseline risk factors of CVD.  ***CMV:*** The data analysis strategy will be to compare the immune risk phenotype parameters in participant with and without CVD, according to HIV and CMV statuses. The significance of between group results will be assessed using t-tests for comparisons of 2 groups or ANOVA for comparisons of multiple groups. Adjusted analysis for cardiovascular risk factors will be obtained using multivariate linear regression. |
| **4 – Characterization of genetic profile in CVD** | The primary outcome will be the presence of and interaction between HIV status and the value of the genetic risk score on the association between genetic risk score and CVD. Linear regression will be used to assess the association between the genetic risk score and presence of subclinical disease, measured as coronary calcium score, coronary plaque volume or carotid intima-media thickness (continuous measures). Models will be adjusted for the following covariates: sex, Framingham risk score (which includes age) and use of secondary prevention therapies (including dichotomous covariates for use of ASA, other anti-platelet agents, anti-hypertensives, statins and antiretrovirals) at time of calcium score, coronary plaque volume or carotid intima-media measurement. Interactions between the genetic risk score and HIV status will be assessed using multiplicative interaction terms in the combined cohort (HIV+ and HIV-). Drug and genetic risk score interactions will be assessed using separate interaction terms for each drug of interest among HIV+ individuals and HIV- individuals separately. Hazard ratios and 95% confidence intervals will be computed. |
| Note: for all analyses, a 2-sided p-value <0.05 will be considered significant. | |
